# Multilevel regulation of muscle-specific transcription factor *hlh-1* during *Caenorhabditis elegans* embryogenesis

**DOI:** 10.1007/s00427-020-00662-9

**Published:** 2020-06-19

**Authors:** Guoye Guan, Meichen Fang, Ming-Kin Wong, Vincy Wing Sze Ho, Xiaomeng An, Chao Tang, Xiaotai Huang, Zhongying Zhao

**Affiliations:** 1 Center for Quantitative Biology, Peking University, Beijing, 100871 China; 2 School of Life Sciences, Peking University, Beijing, 100871 China; 3 Department of Biology, Hong Kong Baptist University, Hong Kong, 999077 China; 4 Peking-Tsinghua Center for Life Sciences, Peking University, Beijing, 100871 China; 5 School of Physics, Peking University, Beijing, 100871 China; 6 School of Computer Science and Technology, Xidian University, Xi’an, 710126 Shaanxi China; 7 State Key Laboratory of Environmental and Biological Analysis, Hong Kong Baptist University, Hong Kong, 999077 China

**Keywords:** Transcription factor, *hlh-1*, Embryogenesis, *Caenorhabditis elegans*, Lineal expression, Multilevel regulation

## Abstract

*hlh-1* is a myogenic transcription factor required for body-wall muscle specification during embryogenesis in *Caenorhabditis elegans*. Despite its well-known role in muscle specification, comprehensive regulatory control upstream of *hlh-1* remains poorly defined. Here, we first established a statistical reference for the spatiotemporal expression of *hlh-1* at single-cell resolution up to the second last round of divisions for most of the cell lineages (from 4- to 350-cell stage) using 13 wild-type embryos. We next generated lineal expression of *hlh-1* after RNA interference (RNAi) perturbation of 65 genes, which were selected based on their degree of conservation, mutant phenotypes, and known roles in development. We then compared the expression profiles between wild-type and RNAi embryos by clustering according to their lineal expression patterns using mean-shift and density-based clustering algorithms, which not only confirmed the roles of existing genes but also uncovered the potential functions of novel genes in muscle specification at multiple levels, including cellular, lineal, and embryonic levels. By combining the public data on protein-protein interactions, protein-DNA interactions, and genetic interactions with our RNAi data, we inferred regulatory pathways upstream of *hlh-1* that function globally or locally. This work not only revealed diverse and multilevel regulatory mechanisms coordinating muscle differentiation during *C. elegans* embryogenesis but also laid a foundation for further characterizing the regulatory pathways controlling muscle specification at the cellular, lineal (local), or embryonic (global) level.

**Electronic supplementary material:**

The online version of this article (10.1007/s00427-020-00662-9) contains supplementary material, which is available to authorized users.

## Introduction

Cell fate specification is precisely and robustly regulated during metazoan development. Cells gain their identities via delicate genetic regulatory networks with spatial and temporal specificity. Such genetic networks usually involve multiple transcription factors that lead to stepwise differentiation by regulating a group of downstream target genes (Davidson 2006). Embryogenesis in *Caenorhabditis elegans* involves rapid cell proliferation and differentiation, resulting in the hatching of the egg into a larva containing 558 cells in about 14 h at room temperature. During embryogenesis, cell division is asymmetric starting from the zygote, leading to blastomeres with different developmental potential, including AB, EMS, E, MS, C, D, P1, P2, P3, and P4. The progenies of blastomeres are then named after their blastomere indicating their division axis or round. For example, E4 denotes E lineage with four progenies, whereas ABal denotes AB granddaughter after two rounds of division first along the anterior-posterior axis (a–p) and then along the left-right axis (l–r). *C. elegans* has been widely used as a model to study cell fate specification and organogenesis at single-cell resolution due to its invariant developmental lineage (Sulston et al. 1983). Several transcription factors that determine the fate of different cell types during *C. elegans* embryogenesis have been identified, such as *hlh-1* for myogenesis and *pha-4* for pharynx formation (Krause et al. 1990; Mango et al. 1994; Shao et al. 2013).

*hlh-1* encodes a transcription factor that belongs to the MyoD family, which functions as a myogenic commitment transcription factor over a broad time window by regulating thousands of genes in *C. elegans* (Krause et al. 1990; Fukushige and Krause 2005; Fukushige et al. 2006; Fox et al. 2007; Bar-Lavan et al. 2016). MyoD family members encode a basic helix-loop-helix transcription factor and serve as early markers of muscle specification in both vertebrates and invertebrates (e.g., mouse and nematode) (Krause et al. 1990; Davis et al. 1987). Worms homozygous for *hlh-1* deficiencies still have normal-appearing body plan and number of muscle cells, but abnormally weak and uncoordinated contraction during late embryogenesis, and eventually fail to hatch (Chen et al. 1992). *hlh-1* starts being expressed mainly in striated muscle cells during early embryonic cell proliferation, but there are also two other expression windows: some descendants of MS turn into other muscle or non-muscle cells at an early stage and six glial-like cells (GLR cells) at a late stage (Chen et al. 1992). HLH-1 protein binds to and regulates numerous well-studied muscle-specific genes and muscle chaperones, contributing to particular proteostasis networks during muscular tissue formation (Bar-Lavan et al. 2016). As the body-wall muscle cells are derived from more than one lineage in *C. elegans* (Sulston et al. 1983) and *hlh-1* is also expressed in a few cells other than those with muscle fate, it is important to infer the upstream regulatory pathways controlling *hlh-1* expression at different levels (e.g., cellular, lineal, and embryonic levels).

The existing *hlh-1* upstream regulatory model suggests that *hlh-1* has a positive feedback loop and self-activation effect, ensuring its continuous expression in body-wall muscle cells during tissue formation (Lei et al. 2009). At the early stage of embryogenesis, *hlh-1* expression is controlled by maternal transcription factors, e.g., *pal-1*, *pop-1*, and *skn-1*, and then by zygotic genes (Lei et al. 2009; Baugh et al. 2005; Hunter and Kenyon 1996; Bowerman et al. 1992). When the POP-1 level is low, high *pal-1* expression triggers C cells to develop into muscle; otherwise, the cells may adopt an epithelial fate, which is mediated through the Wnt/MAP kinase signaling pathway (Fukushige and Krause 2005; Lei et al. 2009). It was also found that *pal-1* regulates *hlh-1* by interacting with its first enhancer in C and D lineages (Lei et al. 2009), whereas another maternal transcription factor, *skn-1*, only affects the development of body-wall muscle cells in MS lineage (Bowerman et al. 1992). It has been reported that RNA interference (RNAi) perturbation of *pal-1* can eliminate *hlh-1* expression in C and D lineages but not in MS lineage, supporting a lineage-based mechanism for muscle fate specification (Baugh et al. 2005). Although ample knowledge has been accumulated on muscle differentiation in *C. elegans*, comprehensive knowledge on the regulation of cell- and lineage-specific *hlh-1* expression is still elusive, particularly with respect to the identity of the underlying direct and indirect regulators of *hlh-1*.

Although it is straightforward and efficient to identify a gene’s downstream targets by high-throughput experimental approaches such as chromatin immunoprecipitation followed by deep sequencing, investigating its upstream regulatory pathways is much more difficult, particularly in vivo and at cellular level. Therefore, apart from experiment-oriented study, it is also necessary and fruitful to use computational approaches to infer upstream regulatory pathways of transcriptional factors based on information from various types of genetic or physical interactions collected from experimental data. Plenty of mathematical methods have been applied for network inference based on available data and different types of interactions. Yeang et al. (2004) proposed a physical network model that uses protein-protein interaction (PPI), protein-DNA interaction (PDI), and gene-knockout data to identify possible signaling pathways in yeast based on the Bayesian network theory (Yeang et al. 2004). Ourfali et al. (2007) suggested an optimized model named signaling-regulatory pathway inference (SPINE) that analyzes the activating or inhibitory effects of genes from gene-knockout data to explain the maximum number of cause-effect pairs (Ourfali et al. 2007). Using *C. elegans* as a model system, several researchers have made great efforts in computational method improvement as well as data-based network reconstruction. Stigler and Chamberlin (2012) inferred a regulatory network for muscle and skin development in *C. elegans* embryos but only focused on C lineage and took a small number of cells into account (Stigler and Chamberlin 2012). Huang et al. (2016, 2017) proposed a framework to infer the signaling networks of the hypodermis-specific transcription factor *nhr-25* at the sublineage level by exploring single-cell expression data obtained from RNAi-perturbed embryos (Huang et al. 2016; Huang et al. 2017). By taking advantage of both molecular interaction information and bioinformatics computational approaches, we attempted to infer the upstream pathways of *hlh-1* and identify the potential candidate genes that regulate it directly or indirectly.

In this study, we first generated a statistical reference for the lineal expression of the muscle-specific transcription factor *hlh-1* at single-cell resolution using 13 wild-type *C. elegans* embryos. We next reconstructed the *hlh-1* expression pattern in mutants of 65 genes (133 embryos), which were selected through its conservation and embryonic phenotype upon perturbation (Ho et al. 2015). To validate our assay, the *hlh-1* promoter-fusion marker system we used was compared with the result reported by Murray et al. (2012) (Murray et al. 2012). We further perturbed and examined two genes with known and expected effects on *hlh-1* expression (*pie-1* and *par-2*). Although a normal *C. elegans* wild-type embryo can generate around 550 cells before the larval stage, we chose the duration up to the second last rounds of divisions in most of the lineages (approximately 350-cell stage) for study, considering that the editing work after this stage would be extremely difficult and time-consuming (Sulston et al. 1983; Ho et al. 2015). In addition, this selected duration covers the processes of *hlh-1* expression initiation and muscular cell specification including some key developmental processes such as gastrulation. Based on the data on PPIs, PDIs, and genetic interactions (GIs) from several public databases along with genes whose perturbation produced deviation of *hlh-1* lineal expression in this study, we inferred the regulatory pathways upstream of *hlh-1* that function globally or locally (MS, C, and D lineages). This study provides a framework for further characterization of the regulatory pathways of *hlh-1* in vivo and facilitates genetic research in body-wall muscle specification.

## Results

### Establishment of lineal expression of *hlh-1* in wild-type embryos

**Fig. 1:**
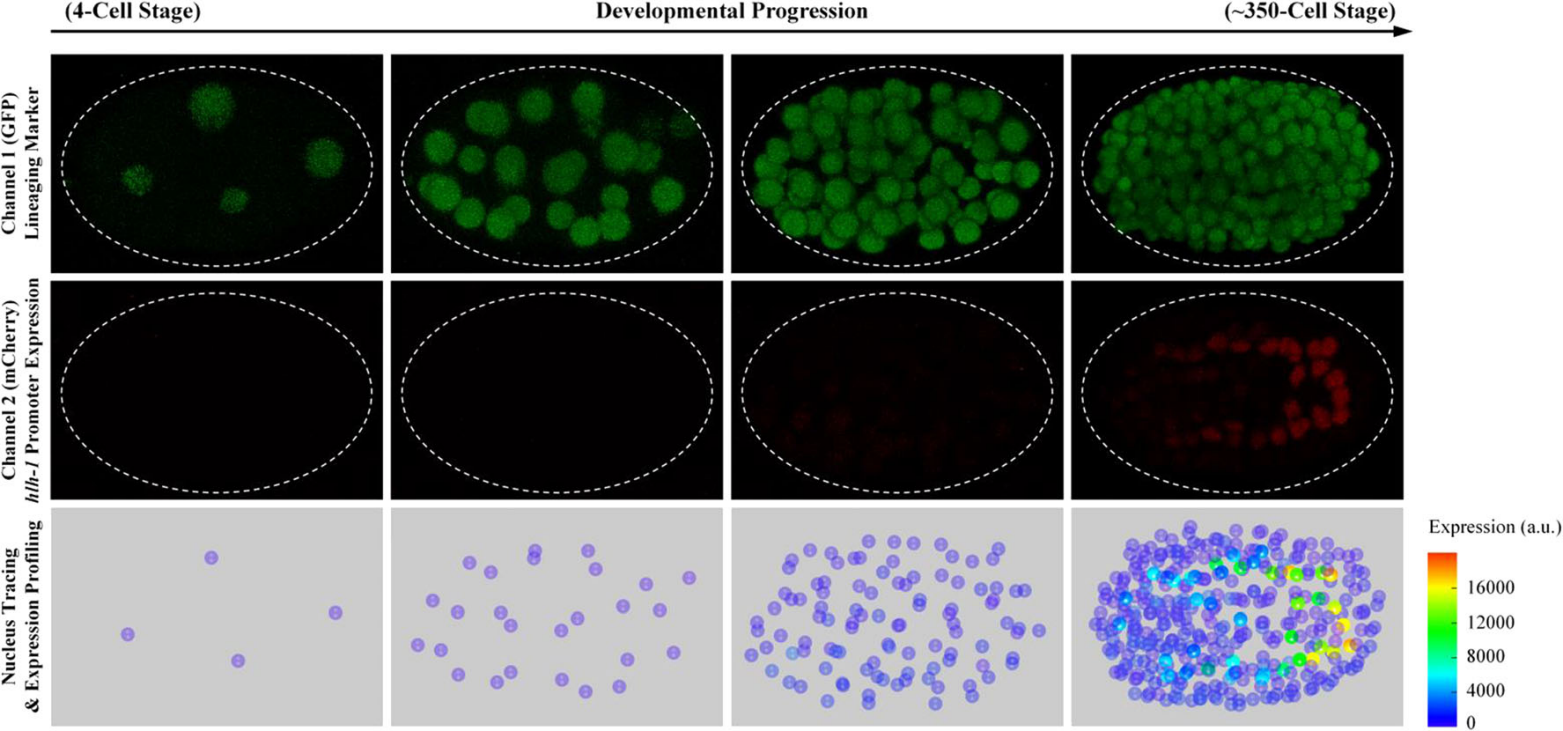
3D time-lapse projections of nuclear expression of GFP (green) for lineage tracing (top), mCherry (red) for *hlh-1* expression profiling (middle), and reconstructed cell positions with expression intensity (bottom) in a *C. elegans* embryo. A white-dashed ellipse is plotted in the experimental images to outline the embryo boundary, as some of the them are invisible due to no mCherry signal. The scale of reconstructed *hlh-1* expression intensity is indicated in the bottom right corner

**Fig. 2:**
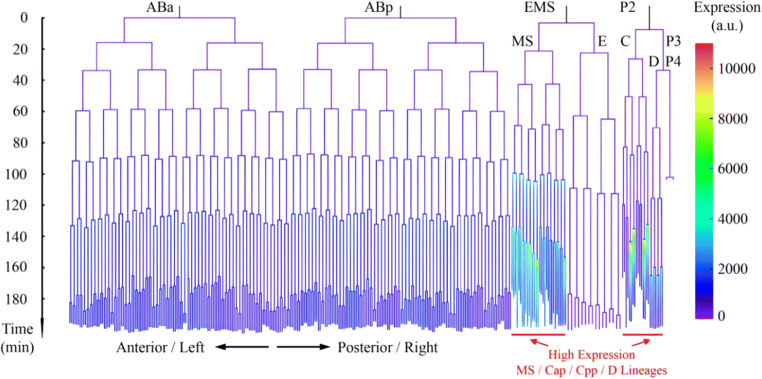
A cell-lineage tree showing lineal expression of *hlh-1* in wild-type embryos from 4- to 350-cell stage. Cell cycle length and expression intensity are normalized and averaged (*n* = 13)

**Fig. 3:**
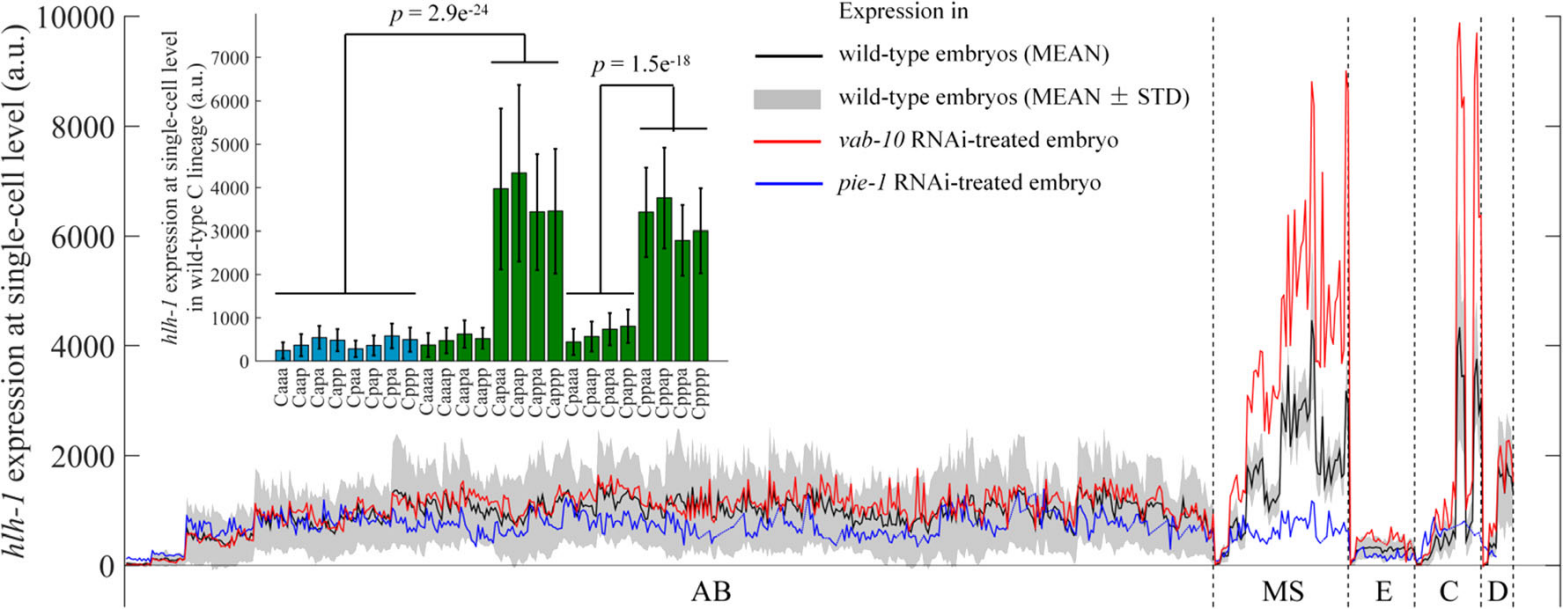
Quantification of *hlh-1* expression in wild-type and RNAi-treated embryos against *vab-10* and *pie-1*. Inset, statistical comparison of *hlh-1* expression between cells in wild-type C lineage (*n* = 13, Wilcoxon rank-sum test)

Based on singe-cell *hlh-1* expression profiling of 13 wild-type embryos, three main groups of high-expressing cells were identified, including MS lineage (MS16, MS32), C lineage (Cap and Cpp sublineages), and D lineage (D8), whereas the remaining lineages, including AB lineage, E lineage, and Caa and Cpa sublineages, showed *hlh-1* expression at background level (Figs. 1 and 2, Tables S1–S3, Supplementary Material 1). Taking C lineage as an example, *hlh-1* expression in Cap and Cpp cells was significantly higher than that in their mothers and sisters, whereas the expressions in Caa and Cpa cells were similar to each other and to that in their mothers at background level (Figs. 2 and 3). In addition, *hlh-1* expression in two subsequent generations of cells in MS lineage showed a gradual increase. In summary, up to the 350-cell stage, cells identified with lineally specific expression of *hlh-1* were as follows: all of the descendants of MS after four rounds of divisions (MSxxxx, MSxxxxx), all of the descendants of Cap and Cpp after two rounds of divisions (Capxx, Cppxx), and all of the descendants of D after three rounds of divisions (Dxxx) (Fig. 2). These together were used to evaluate the global shift in *hlh-1* expression between wild-type and RNAi-treated embryos (Fig. S1). However, there was an extreme outlier in AB lineage, ABprpppppa, which would develop into body muscle but did not exhibit significant fluorescence. The reason for this detection failure is unclear, but it may be owing to the reporter construct’s non-native position and environment. Despite our assay’s disadvantage in activity and sensitivity for ABprppppa, the wild-type embryonic cells expressing *hlh-1* were well consistent with the reported precursors of body-wall muscle cells in MS, C, and D lineages, i.e., all of the body-wall muscle cells were observed to express *hlh-1* except ABprppppa (Sulston et al. 1983). Therefore, we only used the *hlh-1*-expressing cells in P1 lineage (namely, MSxxxx, MSxxxxx, Capxx, Cppxx, and Dxxx) for further investigation. It is worth noting that some cells with non-body wall muscle fate also exhibited high expression. For example, most progenies in MSaa and MSpa lineages would develop pharynx fate (Sulston et al. 1983; Chen et al. 1992), implying that in addition to *hlh-1*, there are other genes and proteins that play a role in the fate determination of MS cells between muscle and pharynx. Interestingly, the expression intensity of *hlh-1* varied between cells and between groups, although they develop into the same terminal body-wall muscle fate (Figs. 2 and 3, Tables S1 and S2). For instance, *hlh-1* expression in MSa sublineage was significantly higher than that in MSp sublineage (*p* value = 1.5203 × 10^−4^) and D lineage (*p* value = 1.0084 × 10^−4^). This indicates that the underlying regulatory pathways in these lineages may be different to some extent, which probably contributes to their subsequent unique cell- and lineage-specific behaviors, including transcription profiling, proliferation rate, migration trajectory, and fate specification (Sulston et al. 1983; Tintori et al. 2016; Packer et al. 2019). To uncover the genes that mediate *hlh-1* expression and drive muscle specification at the cellular, lineal, and embryonic levels, mutant perturbation of cell-specific expression is necessary.

### Screening for regulatory genes controlling *hlh-1* expression through RNAi

Using the *hlh-1*-expressing cells as targets, deviation of global expression in 133 RNAi-treated embryos (65 genes in total) was calculated by proportional linear fitting through comparison with the wild-type averages (Tables 1 and 2, Table S4, Supplementary Material 2). With the *p* value threshold set as 0.05, 22 genes were found to enhance (positive effect) *hlh-1* expression, whereas only 3 genes inhibited (negative effect) *hlh-1* expression. For instance, the overall expression of *hlh-1* after RNAi against *vab-10* and *pie-1* was significantly different from that in the wild-type embryos, suggesting their inhibitory and activating roles, respectively (Fig. 3). The results in the RNAi of *pie-1*, a transcription factor asymmetrically segregated and distributed into stem cells (P1, P2, P3, and P4), validate the previous finding of this gene functioning as a master regulator of both *skn-1* and *pal-1*, indirectly guiding *hlh-1* expression and muscle differentiation with lineage specificity (Hunter and Kenyon 1996)*.* It is worth noting that a number of genes with global effects are related to transcription (e.g., *ceh-20* and *leo-1*), translation (e.g., *nifk-1* and *rrp-1*), and metabolism (e.g., *mrps-17* and *adm-2*) (Lee et al. 2018), and they generally function in cell differentiation and are expected to have non-specific biological roles.

**Table 1:** Genes with embryo-level (global) negative effect (inhibition) on *hlh-1* expression

Globally negative effect	Replicate number	Ratio of expression	Pathway	Function
Wild-type	13	1.00 ± 0.20		
par-1^*^	2	1.50 ± 0.25	Maternal	Exhibits myosin II tail-binding activity, protein serine/threonine kinase activity, and ubiquitin protein ligase-binding activity in human
vab-10^*^	2	1.86 ± 0.41	Cell-cell adhesion	Predicted to have actin filament-binding activity, involved in cell-cell adhesion and epidermis morphogenesis in human
ubc-12^*^	2	1.81 ± 0.63	Protein degradation	Exhibits NEDD8 transferase activity, involved in negative regulation of DNA damage response, signal transduction by p53 class mediator and protein neddylation in human

*p* value: *, 0.05; **, 0.01; ***, 0.001

**Table 2:** Genes with embryo-level (global) positive effect (activation) on *hlh-1* expression

Globally positive effect	Replicate number	Ratio of expression	Pathway	Function
Wild-type	13	1.00 ± 0.20		
pie-1^**^	3	0.28 ± 0.02	Maternal	C-X8-C-X5-C-X3-H-type zinc finger protein
par-2^*^	2	0.62 ± 0.03	Maternal	Protein containing a C3HC4-type RING-finger found in E3 ubiquitin ligase subunits
ceh-20^*^	2	0.57 ± 0.19	Transcription	Contributes to RNA polymerase II regulatory region sequence-specific DNA-binding activity
leo-1^*^	2	0.60 ± 0.06	Transcription	Predicted to encode a protein with the following domain: Leo1-like protein in human. Component of the Paf1 complex, which associates with RNA polymerase II and is involved in histone methylation in *S. cerevisiae*
nifk-1^*^	2	0.67 ± 0.17	Ribosome biogenesis	A nucleolar protein interacting with the FHA domain of MKI67 predicted to have RNA-binding activity in human; constituent of 66S pre-ribosomal particles involved in 60S ribosomal subunit biogenesis in *S. cerevisiae*
rrp-1^*^	2	0.44 ± 0.15	Ribosome biogenesis	RRP1B (ribosomal RNA-processing 1B) exhibits transcription coactivator activity in human
Y94H6A.5^*^	2	0.50 ± 0.02	Ribosome biogenesis	Predicted to have ATP-binding activity, ATP-dependent RNA helicase activity, and RNA-binding activity in human; putative ATP-dependent RNA helicase of the DEAD-box protein family that an essential protein involved in ribosome biogenesis in *S. cerevisiae*
mrps-17^*^	2	0.58 ± 0.14	Metabolism	Mitochondrial ribosomal protein S17 in human
B0035.3^*^	2	0.50 ± 0.06	Metabolism	Exhibits deacetylase activity and hydrolase activity, acting on glycosyl bonds in human
adm-2^*^	2	0.66 ± 0.03	Metabolism	Predicted to have metalloendopeptidase activity in human
C50B6.7^*^	2	0.48 ± 0.05	Metabolism	Predicted to have alpha-amylase activity in human
cogc-2^***^	4	0.55 ± 0.09	Posttranslational modification	Acts as component of the peripheral membrane COG complex that is involved in intra-Golgi protein trafficking in human
F42F12.3^*^	2	0.44 ± 0.15	Posttranslational modification	Predicted to have 3-oxo-5-alpha-steroid 4-dehydrogenase activity in human
algn-11^*^	2	0.56 ± 0.10	Posttranslational modification	Catalyzes sequential addition of the two terminal alpha 1,2-mannose residues to the Man5GlcNAc2-PP-dolichol intermediate during asparagine-linked glycosylation in the ER in *S. cerevisiae*
nra-4^*^	2	0.69 ± 0.03	Unknown	Localized to the endoplasmic reticulum in human
tads-1^*^	2	0.29 ± 0.00	Unknown	Limited homolog to DNA replication termination factor
C32E12.4^*^	2	0.68 ± 0.14	Unknown	Enriched in the AFD, the OLL, the PVD, and the muscle cell
gad-1^*^	2	0.38 ± 0.04	Unknown	WD repeat-containing protein
tbc-2^*^	2	0.51 ± 0.02	Protein transport	Exhibits GTPase activator activity, involved in positive regulation of GTPase activity and positive regulation of early endosome to late endosome transport in human
pitr-3^*^	2	0.62 ± 0.12	Phosphate transport	Predicted to have inorganic phosphate transmembrane transporter activity in human
ced-5^*^	2	0.53 ± 0.12	Apoptosis	Involved in cytoskeletal rearrangements required for phagocytosis of apoptotic cells and cell motility in human
C37H5.5^*^	2	0.60 ± 0.14	Cell-cycle progression	NOC3-like DNA replication regulator in human; subunit of a nuclear complex with Noc2p and pre-replicative complexes in *S. cerevisiae*

*p* value: *, 0.05; **, 0.01; ***, 0.001

**Fig. 4:**
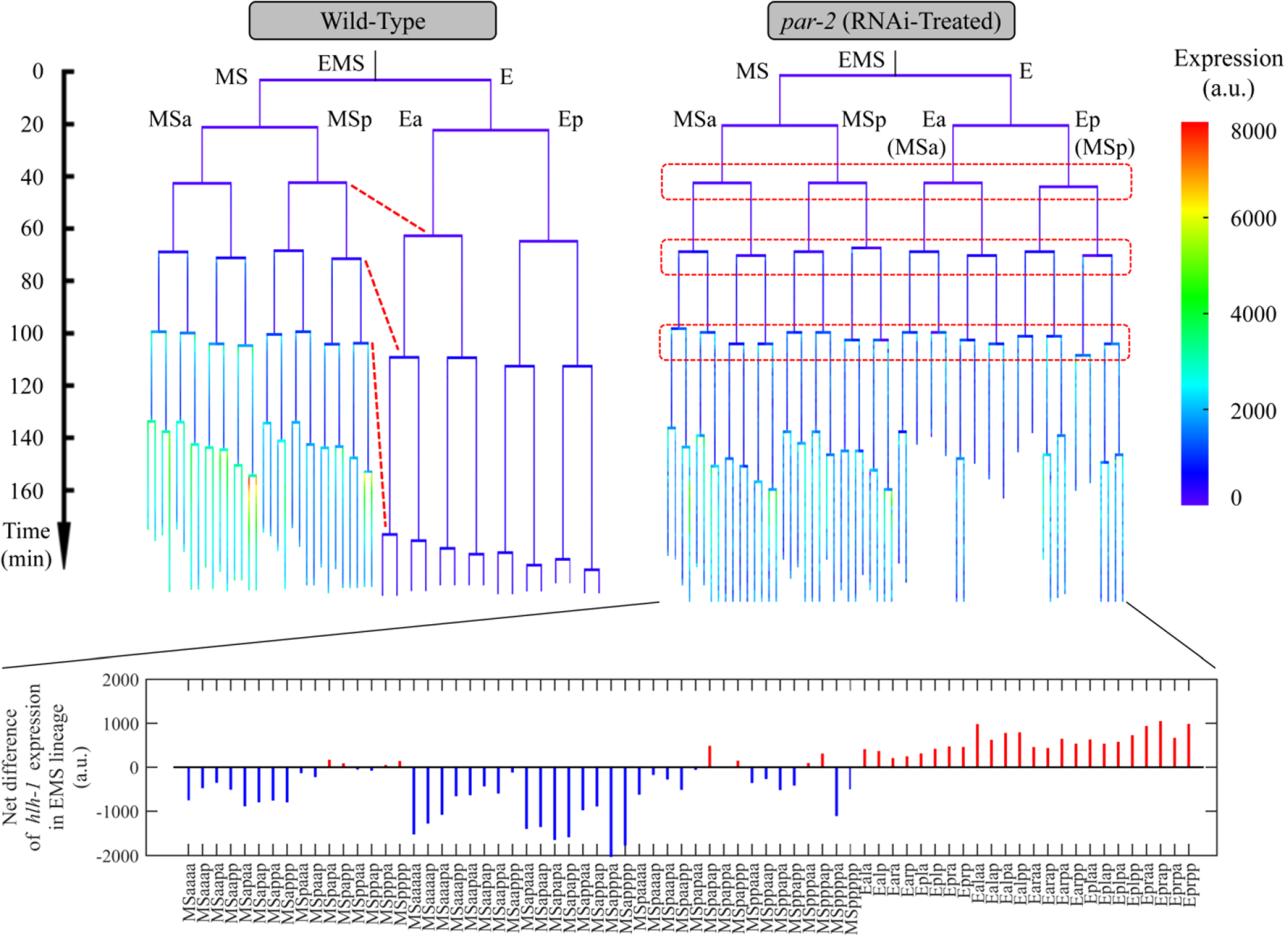
Comparison of cell cycle length and *hlh-1* expression in EMS cells between wild-type and *par-2* RNAi-treated embryos visualized in formats of lineage tree (top) and net difference (bottom)

Cell-cycle asynchrony is another sign of cell differentiation and is usually coupled with fate asymmetry, such as E cells for intestine and MS cells for muscle and pharynx, which are both derived from EMS cells but divide asynchronously as EMS receives Wnt signaling from P2 (Ho et al. 2015; Wong et al. 2016; Thorpe et al. 1997). Intriguingly, Cap and Cpp sublineages, which showed significantly higher *hlh-1* expression than their sisters Caa and Cpa sublineages, also exhibited apparent asynchrony in accordance with their expression asymmetry (Fig. 3). To assess whether a cell with perturbed *hlh-1* expression adopts an altered fate, cell-cycle asynchrony between sister cells could also be evaluated. The maternal transcription factor *par-2* encodes a ring finger protein, PAR-2, involved in fate differentiation, volume segregation, spindle orientation, and cell-cycle progression during early embryogenesis, which establishes cell polarity in cooperation with other proteins from the PAR and MEX families (Goldstein and Macara 2007; Nance 2010; Hubatsch et al. 2019). As expected, as the progenitor of E and MS cells lost polarity and asymmetry, these two groups of cells are divided synchronously upon RNAi against *par-2*; consequently, the *hlh-1* expression in E cells was enhanced and that in MS cells was reduced (Fig. 4). These opposite changes in *hlh-1* expression in E and MS cells suggest the presence of differentiated regulatory networks upstream of *hlh-1* in these cells.

### Phenotype clustering of genes regulating *hlh-1* expression

The overall test method could uncover the global impact of genes but may have masked the specific causal link at the cellular level. Therefore, we further used the Wilcoxon rank-sum test to estimate whether there is a positive or negative effect on *hlh-1* expression in each cell, finally generating a cell-level binary-coded phenotype for each gene (Supplementary Material 3).

**Fig. 5:**
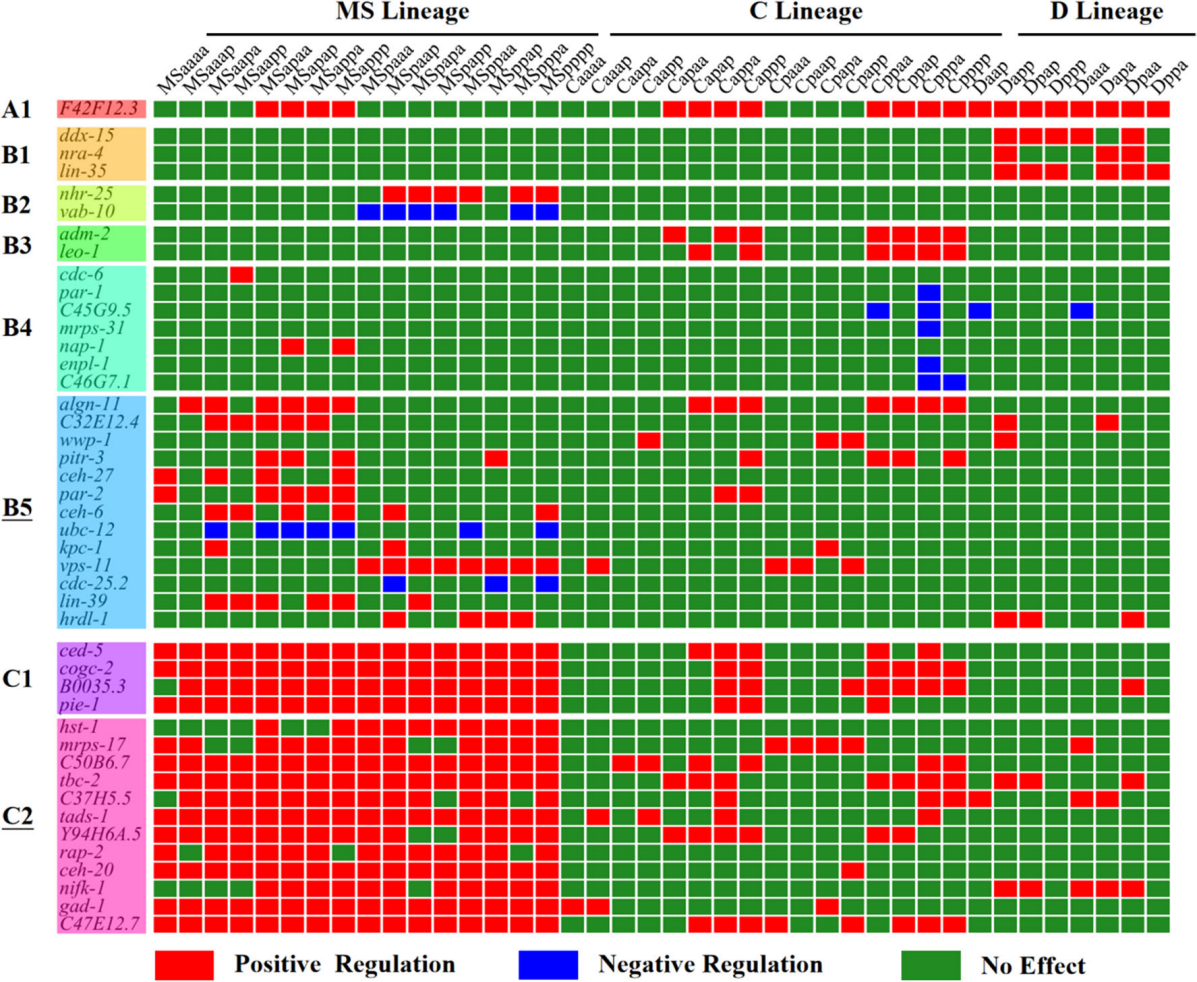
Clustering of genes with lineage-specific effects on *hlh-1* expression based on significant deviation in expression intensity from that of wild type at single-cell resolution after RNAi (*n* ≥ 2, Wilcoxon rank-sum test, *p* value < 0.05)

According to the single-cell binary phenotypes of mutants, all of the genes with significant effects on the considered MS16, C16, or D8 cells were clustered (Fig. 5). Here, two clustering methods were performed successively. First, all of the genes were divided into three categories (**A**, **B**, and **C**) using the mean-shift algorithm (Comaniciu and Meer 2002), which could handle a complex multimodal feature space and separate the objects based on their overall characteristics (affected cells/cell groups). The first group (**A1**) contains only one gene (F42F12.3), which precisely and specifically enhances *hlh-1* expression in the progeny cells of MSap, Cap, Cpp, and D, while the others remain unchanged. The second group (**B1**–**B5**) contains genes that affect *hlh-1* expression in a relatively small fraction of cells, whereas the third group (**C1**-**C2**) has a general positive effect on MS lineage.

Next, the Density-Based Spatial Clustering of Application with Noise (DBSCAN) (Ester et al. 1996) was applied to each category for subtle feature extraction, and the unclassified genes were labeled as “noise” genes (**B5**, **C2**), which are relatively far from all other genes. After removing the “noise” genes, a total of five more clusters (**B1**, **B2, B3**, **B4**, and **C1**) were obtained, and phenotypic patterns within each cluster could be clearly described and interpreted as below:

- **B1**: *ddx-15*, *nra-4*, and *lin-35* have a positive effect on *hlh-1* expression, specifically in D8 cells.
- **B2**: *nhr-25* and *vab-10* have a positive and negative effect, respectively, specifically in MSp progenies.
- **B3**: *adm-2* and *leo-1* have a positive effect on *hlh-1* expression, specifically in Cap and Cpp progenies.
- **B4**: *cdc-6*, *par-1*, C45G9.5, *mrps-31*, *nap-1*, *enpl-1*, and C46G7.1 have no obvious common rules but only affected a few cells, particularly Cpppa cells in which *hlh-1* expression was regulated negatively.
- **C1**: *ced-5*, *cogc-2*, B0035.3, and *pie-1* have a general positive effect on *hlh-1* expression in MS lineage and Cap and Cpp sublineages.

Note that within the two “noise” groups that have comparatively weaker relation to the others, the genes have much more diverse expression patterns, with affected cells distributed in different lineages and sublineages, possibly contributing to the specific developmental behaviors of different cells as well.

With the clustering results, three major types of genes with specificity at different scales were identified: functioning in a few targeted cells (**B4**), in only one lineage/sublineage (**B1** and **B2**), and in multiple lineages/sublineages (**A1**, **B3**, **B5**, **C1**, and **C2**). Given the invariant development of *C. elegans* (Sulston et al. 1983), *hlh-1* expression is accurately regulated at multiple levels, including cellular, lineal, and embryonic levels, which may coordinate with one another to precisely regulate the development of the multicellular animal.

In addition to phenotypes at the cellular and embryonic (global) levels, regulation at the lineal level (i.e., with lineage specificity) also deserved attention (Fig. 5). For the three lineages with high *hlh-1* expression (MS, C, and D), if a gene exerted an effect on more than half of the cells in the generation in which *hlh-1* was first expressed (8 for MS16, 4 for C16, and 4 for D8), this gene would be regarded as having general effects on this lineage. Accordingly, 10 genes were found to have such effects in MS lineage (C37H5.5, *ceh-20*, *mrps-17*, *pie-1*, *gad-1*, *tads-1*, *nifk-1*, *cogc-2*, *tbc-2*, and *ced-5*), 6 in C lineage (*cogc-2*, *tbc-2*, *ced-5*, *leo-1*, *adm-2*, and F42F12.3), and 4 for D lineage (*nifk-1*, F42F12.3, *lin-35*, and *ddx-15*) (Table 3). These genes probably contribute to lineage-specific transcriptome, proteome, and morphological behavior. Whether they have molecular interaction with and respond to the known regulators (e.g., *skn-1*, *pop-1*, and *pal-1*) needs further verification. It is worth noting that several genes with global regulation were not identified here, because the number of cells that they affected was below the arbitrary criterion. For example, in the RNAi embryos against the three genes (*ubc-12*, *par-1*, and *vab-10*) that exhibited inhibition, no cell acquired weaker *hlh-1* expression, and at least four cells exhibited significantly stronger *hlh-1* expression after RNAi perturbation, making them notable as global effectors, but the numbers of their affected cells were below the selection criterion (Fig. 5).

**Table 3:** Genes with lineage-level (local) effect on *hlh-1* expression (+, activation)

Gene	MS	C	D
C37H5.5	+		
*ceh-20*	+		
*mrps-17*	+		
*pie-1*	+		
*gad-1*	+		
*tads-1*	+		
*nifk-1*	+		+
*cogc-2*	+	+	
*tbc-2*	+	+	
*ced-5*	+	+	
*leo-1*		+	
*adm-2*		+	
F42F12.3		+	+
*lin-35*			+
*ddx-15*			+

### Inference of upstream regulatory networks of *hlh-1*

**Fig. 6:**
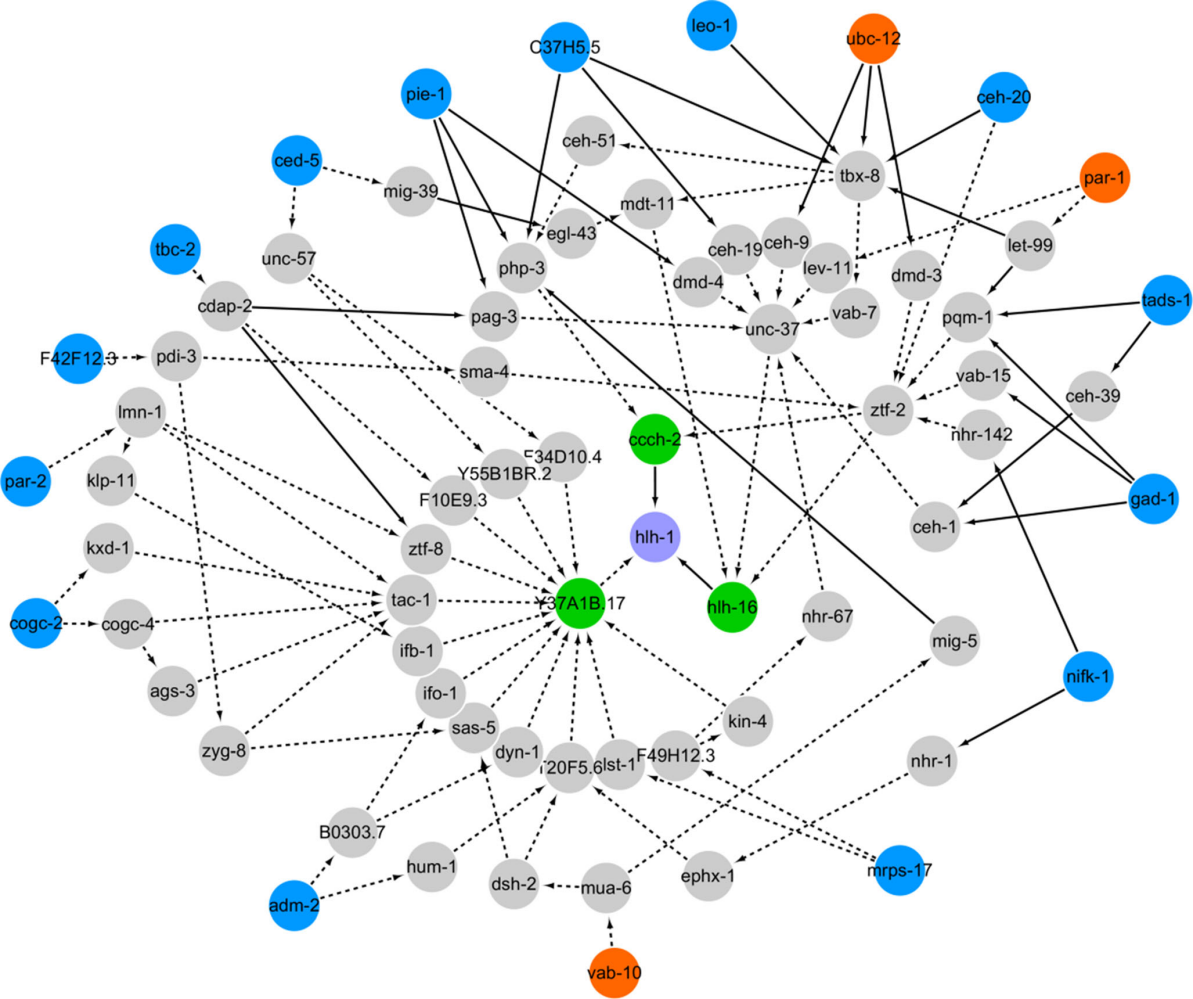
Regulatory pathways upstream of *hlh-1* in embryo, inferred using PPI, PDI, GI, and lineaging data before and after RNAi. Purple circle denotes the terminal gene *hlh-1* of the whole signaling network; green circles denote the upstream genes that physically interact with *hlh-1*; blue and orange circles denote the knocked-down genes with positive and negative regulations on *hlh-1*, respectively; black lines with arrow represent the direction of pathways inferred from PDI (solid line) or PPI (dashed line) data

To further delineate the regulatory mechanism of *hlh-1*, genes with significant embryo-level or lineage-level effects on *hlh-1* expression in MS, C, and D lineages were subjected to pathway inference and network construction. Subsequently, the three most probable pathways of each gene were selected and visualized using Cytoscape software (Table S5) (Shannon et al. 2003). As the known genetic regulation information is limited, it was not possible to completely deduce whether an interaction is positive or negative in many cases. According to the screening assay and results on embryo-level and lineage-level regulation, one global network and three local networks were reconstructed (Fig. 6, Figs. S4-S6). All of the pathways converged to three nodes connected to *hlh-1*: *ccch-2*, *hlh-16*, and Y37A1B.17. The interaction between *ccch-2* or *hlh-16* and *hlh-1* was found to be a PDI, whereas that between Y37A1B.17 and *hlh-1* was found to be a PPI. Some previous studies on network inference have suggested that the interaction directly linked to the target gene should be a PDI for direct, strong, and reliable regulation (Stigler and Chamberlin 2012). However, considering the self-activation function of the HLH-1 protein (Lei et al. 2009), that restriction was excluded here.

Limited data on the three key genes identified here are available from previous works. *ccch-2* is a transcription factor with metal ion-binding ability. Proteins in the same family (tandem zinc finger family) play roles in the development from oocyte to embryo (e.g., polarity formation). However, the detailed developmental function of *ccch-2* is poorly understood (Kaymak et al. 2010). Besides, *hlh-16* was proposed to be left-right asymmetrically expressed in different lineages of nervous systems (AB lineage), and its asymmetric pattern was proposed to be established during gastrulation (Bertrand et al. 2011). Both *ccch-2* and *hlh-16* were highly expressed in C and MS lineage, respectively, and are likely involved in the regulation of *hlh-1* and muscle specification in these two cell groups (Hashimshony et al. 2014). Meanwhile, Y37A1B.17 is a homolog of the human dynamin-binding protein, and its human homolog, Tuba, is a scaffold protein that links the dynamin and actin regulatory proteins (Salazar et al. 2003). The inferred network showed that Y37A1B.17 interacts with several nodes, which may be due to false-positive detection of PPIs in the experiments (Phizicky and Fields 1995). Although the interaction data have been weighted based on reliability of different experimental methods, the quality of the original data on the background network heavily affects the computational results.

Among the hubs inferred to indirectly regulate *hlh-1*, some genes have been reported to be correlated with muscle tissue formation, such as *sma-4*, *unc-37*, and *unc-57*. Recent research on adaptive body physique showed that compared with the young *C. elegans* adults grown in agar culture, those grown in liquid culture have a significantly longer body and exhibit higher expression of *hlh-1* and its downstream target *myo-3*, and that *sma-4*, a well-known transcription factor of the TGF-*β* signaling pathway, plays a dominant role in body length control (Harada et al. 2016). However, how the TGF-*β* signaling pathway modulates muscle gene expression is yet to be understood, and our inference provides a potential candidate pathway for this question. Moreover, the alteration in response to different cultures requires environment-responsive neuromuscular signaling (e.g., *unc-8*). Whether *unc-37* and *unc-57* also function in *hlh-1* expression coordination by involvement in neuromuscular signaling is worth further investigation (Harada et al. 2016; Winnier et al. 1999; Schuske et al. 2003).

## Discussion

Every cell acquires a unique identity and specific fate during the development of *C. elegans*, but the underlying mechanism regulating their development remains to be thoroughly understood. Focusing on the muscle-specific transcription factor *hlh-1*, we first rebuilt the statistical expression profiling reference at single-cell resolution using 13 wild-type embryos. We next generated 133 RNAi-treated embryos for 65 genes in total and quantified the changes in *hlh-1* expression after perturbation. By comparing *hlh-1* expression between wild-type and RNAi-treated embryos using different statistical methods, characteristics at the cellular, lineal, and embryonic levels were quantified and analyzed, providing new insight into gene function during embryonic development. To identify the genes that regulate *hlh-1* expression, their cellular phenotypes were clustered, revealing eight groups of genes with distinct and different regulatory patterns. Finally, we inferred the upstream regulatory pathways and networks using a combination of our RNAi data and existing data on PPIs, PDIs, and GIs. Taken together, the combination of our RNAi mutant screening and the subsequent network reconstruction uncovered dozens of candidate genes that regulate *hlh-1* directly and indirectly.

Different cells and lineages have varying *hlh-1* expression intensity; meanwhile, they can also exhibit differential responses to the same genetic perturbation (e.g., EMS lineage, RNAi against *par-2*, Fig. 4), indicating differential underlying networks between them, in line with the lineage-based mechanisms established before (Lei et al. 2009; Baugh et al. 2005; Hunter and Kenyon 1996; Bowerman et al. 1992). A few genes were selected to uncover their specificity on *hlh-1* regulation, suggesting a set of layer-by-layer and cross-talking regulations during body-wall muscle development in *C. elegans*. Three enhancers related to *hlh-1* are active in different cell lines (Lei et al. 2009), so genes and proteins may cooperate and perform cell- and lineage-specific regulation by acting on different enhancers or their regulatory factors. Further studies on the upstream binding positions of *hlh-1* could provide more insight into the upstream regulation of this gene.

Although dozens of upstream regulators were identified based on their direct or indirect interaction with *hlh-1* and high possibility of such interaction, the inferred pathways still need verification in vivo, especially for the three closest genes (i.e., *ccch-2*, *hlh-16*, and Y37A1B.17), so as to exclude the possibility of false positives and computation bias. One can perform mutation of both the direct and indirect candidate genes and examine the *hlh-1* expression in embryos (Fig. 6, Table S5). In addition, other genes with functions or pathways related to the effective genes (e.g., posttranslational modification) reported in this work are also worth exploring for similar mutant phenotype and correlation to muscle specification. Relative to the whole genome of *C. elegans*, the 65 genes perturbed in this work are limited. It is possible that some other genes could also affect *hlh-1*, particularly those with cell- or lineage-specific expression, which can be selected from a broader gene pool and tested by RNAi experiments. Furthermore, other muscle-specific markers/reporters can be introduced to validate the upstream coordinators of *hlh-1*, such as *myo-3*, a myosin heavy-chain gene that is a target of the HLH-1 protein and also widely used for studying muscle differentiation (Fukushige and Krause 2005; Fukushige et al. 2006; Meister et al. 2010; Gonzalez-Sandoval et al. 2015).

As the myogenic transcription factor *hlh-1* governs the establishment of muscle-specific proteostasis by binding to the promoters of chaperone genes and subsequently regulating the folding of muscle proteins, using it as a gene expression reporter and introducing genetic perturbation in its expression pattern can provide comprehensive information on the regulatory network involved in muscle specification (Bar-Lavan et al. 2016). However, given the lost fluorescence signal for ABprpppppa, our assay is not perfect, and other types of strains (e.g., protein fusion) and markers/reporters (e.g., *myo-3*) can be taken into consideration for capturing more precise muscle-specific gene expression profiles in both wild-type and RNAi-treated embryos (Murray et al. 2012; Fire et al. 1998).

Mutant screening and pathway inference cannot reconstruct or represent the complete details of native regulation, as there could be other genes directly or indirectly regulating *hlh-1*, which were not included in our experiment or public databases. It is also worth of noting that there might be some false-positive or false-negative results from PPI and PDI data, leading to our over or underestimate of the regulatory genes. Experimental validation is necessary before detailed characterization of individual interaction. Considering the fact that the known *hlh-1* regulatory network can sensitively respond to not only the persistent expression level on average but also the transient changes such as a burst in *hlh-1* and *hnd-1*, it is worth investigating the temporal expression phenotype in mutants to uncover more information and details of this system (Fukushige et al. 2006; Gonzalez-Sandoval et al. 2015). Our work not only reveals diverse and multilevel regulatory mechanisms coordinating differentiation during *C. elegans* embryogenesis but also provides novel targets controlling muscle-fate specification in different lineages, sublineages, and cells for further developmental study. This research approaches can be readily applied to other genes or proteins for inference of gene- or pathway-specific regulatory network.

## Materials and methods

### Data collection and preprocessing

Embryo curation, automated lineaging, and single-cell expression profiling were performed as described previously (Ho et al. 2015; Murray et al. 2012; Murray et al. 2006). The strain we used (strain RW10112) expresses a transgenic fusion between *hlh-1* promoter and mCherry from 28-cell stage to over 350-cell stage and specifically labels the muscle fate at single-cell level (Ho et al. 2015). It is expected to contain multiple copies of the fusion, i.e., pJIM20::*hlh-1* construct. To build the construct, the 3147-bp genomic region including the *hlh-1* promoter region and 5′-UTR was amplified and fused with an H1::mCherry reporter (Fig. S7) (Murray et al. 2012). The *hlh-1* expression profiling was generated at the cellular level and then compared with the profile reported previously (Murray et al. 2012), revealing high coincidence in all of the lineages (Fig. S3).

A total of 13 wild-type and 133 RNAi-treated embryos (65 genes and at least two replicates for each), which ubiquitously expressed nuclear green fluorescent protein (GFP) driven by histone promoter and mCherry in body-wall muscle tissue driven by the *hlh-1* cell fate marker, were imaged up to approximately 350-cell stage using time-lapse three-dimensional (3D) confocal microscopy at about 1.5-min intervals (Tables S3 and S4). Cell movement, division timing, and fluorescence intensity were quantitatively measured after automated lineaging. Gene expression profiling was generated to acquire spatiotemporal dynamics of *hlh-1* expression in each cell (Fig. 1, Supplementary Materials 1 and 2). After manual correction for editing errors, cells of AB4, AB8, AB16, AB32, AB64, AB128, MS1, MS2, MS4, MS8, MS16, E1, E2, E4, E8, C1, C2, C4, C8, D1, D2, D4, P3, and P4 in wild-type embryos were confirmed to have their full lifespans recorded, and all of the daughters of AB128, MS16, E8, C8, and D4 were curated for an extra 5.5 min (the exception was Z2/Z3, the germline cells which are derived from P4 and do not divide until the larval stage) (Sulston et al. 1983; Hubbard and Greenstein 2005). These durations were adopted for examination of *hlh-1* expression in all of the cells in both wild-type and RNAi-treated embryos to form a complete and comparable framework with little bias. For each cell, 13 temporal sequences of *hlh-1* expression within its examination range were obtained from wild-type embryos for building a statistical reference and detecting mutant defects. Although cell type and expression data could be obtained from all of the wild-type embryos, this may not be the case for some RNAi embryos due to the unpredictability of defects after RNAi. Therefore, we traced the RNAi-treated embryos to 350-cell stage as much as possible, although some other cells may have been missed (Ho et al. 2015). It is worth pointing out that those 65 perturbed genes were a part of genes we investigated previously, which were prioritized based on their degree of conservation, reported phenotypes, and known roles in development (Ho et al. 2015). Although information on cell division timing and cell identity annotation has been previously published, this is the first study to reveal single-cell gene expression data in these embryos.

### Statistical evaluation of expression perturbation

After building a statistical reference for *hlh-1* expression at single-cell resolution, we next evaluated the activation timing and identity of cells with a significantly higher expression than the background. Using the Wilcoxon rank-sum test, cell lineages or sublineages with expression intensities that were significantly higher than those in other cousin lineages (*p* value < 0.05) were defined as the (sub)lineages that express *hlh-1* along with their daughters.

Based on the list of identified expressing cells, the expression intensities of each RNAi-treated embryo were used for proportional linear fitting to the corresponding averages of the 13 wild-type embryos, producing a slope (ratio) that revealed their global shift from the normal expression level (Fig. S1). Subsequently, the slopes between wild-type and RNAi-treated embryos were analyzed using the Wilcoxon rank-sum test (*p* value < 0.05) to assess whether a gene had a global enhancing or inhibitory effect on *hlh-1* expression. The same test was also conducted to differentiate each cell’s expression between wild-type and RNAi-treated embryos and classify cell-specific regulatory genes. For the lineages that expressed *hlh-1* in wild-type embryos, if a gene exerted an effect on at least half of the cells in a generation, it was considered to have uniform effects on this lineage. Note that for both wild-type and RNAi-treated embryos, the whole temporal expression sequences of cells with complete lifespan (AB1–AB128, MS1–MS16, E1–E8, C1–C8, D1–D4, P3, and P4) were used for calculation and searching, whereas for their daughters (AB256, MS32, E16, C16, and D8), the expression data of only the duration common between the 13 wild-type embryos (more than 5.5 min) were taken into account, because manual editing at a later stage would be impractical.

### Phenotype clustering and feature extraction

After phenotyping based on cell-level expression, genes were clustered according to their *hlh-1* expression patterns at the cellular level (affected or not), starting with the cells of the first generation that started expressing *hlh-1* in each lineage. The effect on a cell was binarized into all or none, regardless of the positive or negative regulation or the degree of influence. To minimize the impact of noise and extract the characteristics of expression in perturbed embryos, the genes were first automatically divided into several categories using the mean-shift algorithm to distinguish their overall differences in phenotype (Comaniciu and Meer 2002). Subsequently, the density-based clustering algorithm DBSCAN was applied to each category for further grouping and feature refinement (Ester et al. 1996).

### Inference of the upstream regulatory network

Great efforts have been made by several research groups to construct and maintain databases of biological interactions (e.g., PPIs, PDIs, and GIs) in different organisms (Salwinski et al. 2004; Licata et al. 2011; Orchard et al. 2012; Chatr-Aryamontri et al. 2014; Orchard et al. 2014). With the accumulation of published articles and data, the IMEx consortium was found to provide a non-redundant set of protein interactions with common data formats (Orchard et al. 2012). In total, 10,392 PPIs from IMEx (Version 2019-02-11) were extracted for this work. In addition, 21,714 PDIs as well as their statistical *Z*-scores were adopted from a study (Fuxman et al. 2016) that used enhanced yeast one-hybrid assays for high-throughput gene-centered detection of PDIs, including 90% of transcription factors and 15% of protein-coding genes (promotors), in *C. elegans* (Fuxman Bass et al. 2016). Moreover, 7823 cause-effect pairs with either activating or inhibitory regulation were obtained from our previous work (Huang et al. 2017), which were originally retrieved from GIs and gene regulatory effect information on WormBase (Version WS248, https://wormbase.org/about/userguide#3%2D%2D10) (Huang et al. 2017; Lee et al. 2018). Several physical, molecular biological, and genetic approaches have been applied to detect PPIs, and each has its specific limits and advantages. For example, in two-hybrid systems, PPIs occur within the native environment of cells, and thus, these systems enable extensive screening, but they have a relatively high false-positive rate. In contrast, protein affinity chromatography is sensitive, but it may occasionally fail to detect interactions, and its results may be inconsistent with those of other approaches (Phizicky and Fields 1995). Here, we used the previously proposed MIscore system to evaluate the reliability of measurement methods and PPIs (Meister et al. 2010). In the MIscore system, the observation method, interaction type, and number of published reports are considered and used for score estimation, which is eventually normalized to a value between 0 and 1 (Villaveces et al. 2015). It is worth noting that the scores of PDI data are usually found to be higher than those of PPI data due to their totally different scoring systems (Fuxman Bass et al. 2016; Villaveces et al. 2015). Despite this difference, both types of data were adopted and used in this work to highlight the superiority of PDI data over PPI data as PDIs are much more direct and reliable regulatory interactions (Yeang et al. 2004).

With effective genes as starting points, we next aimed at exploring all of the pathways connected to the terminal target *hlh-1* on the background network constructed with PPIs and PDIs, using a framework we proposed previously (Huang et al. 2016; Huang et al. 2017). First, the acquired PPI and PDI data were merged into a unified background network constructed using genes as vertexes and their interactions as edges (Fig. S2). To simplify the problem, it is reasonable to assume that each interaction is independent, so that we can apply a Markov chain model, and that the scores of the pathways are simply the products of each edge’s score. Note that the sums of all interactions from one vertex to its neighbors were normalized to 1 and only the pathways with length less than 5 steps were under consideration. Here, {*e*_*i*_} denotes the collection of all edges on a single pathway; *w*_*j*_ denotes the score of the *j*^th^ interaction edge; and *i*_min_ and *i*_max_ denote the initial and terminal edges, respectively. The total score ${W}_{\left\{{e}_i\right\}}$ could then be obtained as follows (Eq. (1)):

1 $$ {W}_{\left\{{e}_i\right\}}=\prod \limits_{i_{\mathrm{min}}\le j\le {i}_{\mathrm{max}}}{w}_j $$

The top three pathways with the highest *W* scores were selected to further infer the most likely regulatory relationship between genes. Information from cause-effect pairs (GIs, RNAi; activation or inhibition) was then integrated to infer the possible direction and the sign of interactions in the pathways. To this end, a potential function *Φ* was designed to estimate whether a configuration of the pathway aligns with a known cause-effect relationship (Eq. (2)). Here, *x*_*j*_ is assigned binary values of 1 for physical interaction existence and 0 for inexistence. *d*_*j*_ is the variable describing the regulatory direction of the *j*^th^ interaction; if the direction is consistent with the interaction, then *d*_*j*_ is 1, otherwise *d*_*j*_ = 0; similarly, *s*_*j*_ is the variable representing the regulatory form of the *j*^th^ interaction; when the interaction leads to positive regulation, then *s*_*j*_ = + 1; in case of negative regulation, *s*_*j*_ = − 1; and in case of no effect, *s*_*j*_ = 0; *k*_(*m,n*)_ denotes the cause-effect pair of vertex *m* and vertex *n*. The indicator function *I* is assigned 1 when the sign combination {*s*_*j*_} accords with the known cause-effect regulation, otherwise 0. Consistently and respectively, {*x*_*j*_}, {*d*_*j*_}, {*s*_*j*_}, and {*k*_(*m,n*)_} denote the set of their variables within a selected path {*e*_*i*_}:

2 $$ {\varPhi}_{\left\{{e}_i\right\}}\left(\left\{{x}_j\right\},\left\{{d}_j\right\},\left\{{s}_j\right\}\left|\left\{{k}_{\left(m,n\right)}\right\}\right.\right)=\prod \limits_{i_{\mathrm{min}}\le j\le {i}_{\mathrm{max}}}{x}_j\cdot \prod \limits_{i_{\mathrm{min}}\le j\le {i}_{\mathrm{max}}}{d}_j\cdot \prod \limits_{k_{\left(m,n\right)}\in \left\{{k}_{\left(m,n\right)}\right\}}\left[I\left(\prod \limits_{m\le j\le n}{s}_j={k}_{\left(m,n\right)}\right)\right] $$

The potential function *Φ*, which ends up outputting 1 for the reasonable pathways and 0 for the unreasonable ones, further maps the experimental cause-effect constraints (activation or inhibition) into the final network configuration. By taking advantage of the molecular interaction information and the abovementioned bioinformatics approaches, we attempted to infer the upstream pathways and networks of *hlh-1* and identify the potential candidate genes with direct or indirect regulation.

## Electronic supplementary material

ESM 1 (DOCX 1.48 mb)
