# Supplementary material for: Multilevel regulation of muscle-specific transcription factor hlh-1 during Caenorhabditis elegans embryogenesis
Source: Dev Genes Evol. 2020 Jun 19;230(4):265–78. doi: 10.1007/s00427-020-00662-9 (PMC7371654; doi:10.1007/s00427-020-00662-9)
Supplement: Supplementary file 1 — (DOCX 1.48 mb) [file 427_2020_662_MOESM1_ESM.docx]

**Figure S1**. Global shift in *hlh-1* expression intensity between individual wild-type embryos and their average. Each dot represents one specific cell that showed a significantly higher *hlh-1* expression (including MSxxxx, MSxxxxx, Capxx, Cppxx and Dxxx) than the remaining cells. Red points indicate the wild-type embryo sample with globally the highest expression; blue dots indicate the wild-type embryo sample with globally the lowest expression.

**Figure S2**. Flow chart of regulatory upstream network inference using PPI, PDI, GI and RNAi data.

**Figure S3**. Side-by-side comparison of *hlh-1* lineal expression from 4 to 350-cell stage for 13 wild-type embryos (top, this study) and a wild-type embryo from a previous study (bottom) (Murray et al. 2012). Cell cycle length and expression intensity are normalized and averaged.

**Figure S4**. Regulatory pathways upstream of *hlh-1* in MS lineage, inferred using PPI, PDI, GI and lineaging data before and after RNAi. Purple circles denote the terminal gene *hlh-1* of the whole signaling network; green circles denote the upstream genes that physically interact with *hlh-1*; blue circles denote the knocked down genes with positive regulations on *hlh-1*, respectively; black lines with arrow represent the direction of pathways: solid lines for PDIs and dashed lines for PPIs.

**Figure S5**. Regulatory pathways upstream of *hlh-1* in C lineage, inferred using PPI, PDI, GI and lineaging data before and after RNAi. Purple circles denote the terminal gene *hlh-1* of the whole signaling network; green circles denote the upstream genes that physically interact with *hlh-1*; blue circles denote the knocked down genes with positive regulations on *hlh-1*, respectively; black lines with arrow represent the direction of pathways: solid lines for PDIs and dashed lines for PPIs.

**Figure S6**. Regulatory pathways upstream of *hlh-1* in D lineage, inferred using PPI, PDI, GI and lineaging data before and after RNAi. Purple circles denote the terminal gene *hlh-1* of the whole signaling network; green circles denote the upstream genes that physically interact with *hlh-1*; blue circles denote the knocked down genes with positive regulations on *hlh-1*, respectively; black lines with arrow represent the direction of pathways: solid lines for PDIs and dashed lines for PPIs.

**Figure S7**. Screenshot of the BLAST result in genome browser from WormBase using the cloned *hlh-1* promoter region as a query. The “hit” is labelled as a thick cyan bar and indicated with a double headed arrow, demarcating the boundaries of the *hlh-1* promoter region. Note that the “hit” covers the last exon of the neighboring gene, *cyp-23A1* to maximize the coverage of the intergenic region between *cyp-23A1* and *hlh-1*.

**Figure S1**


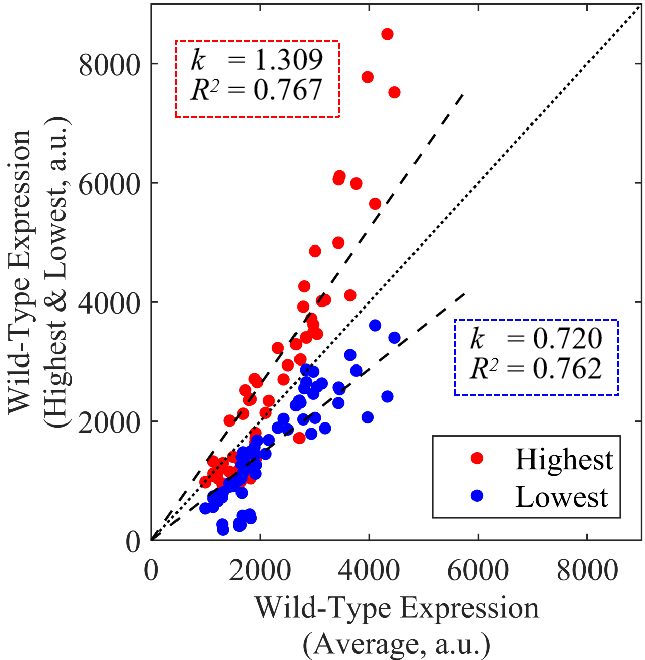


**Figure S2**


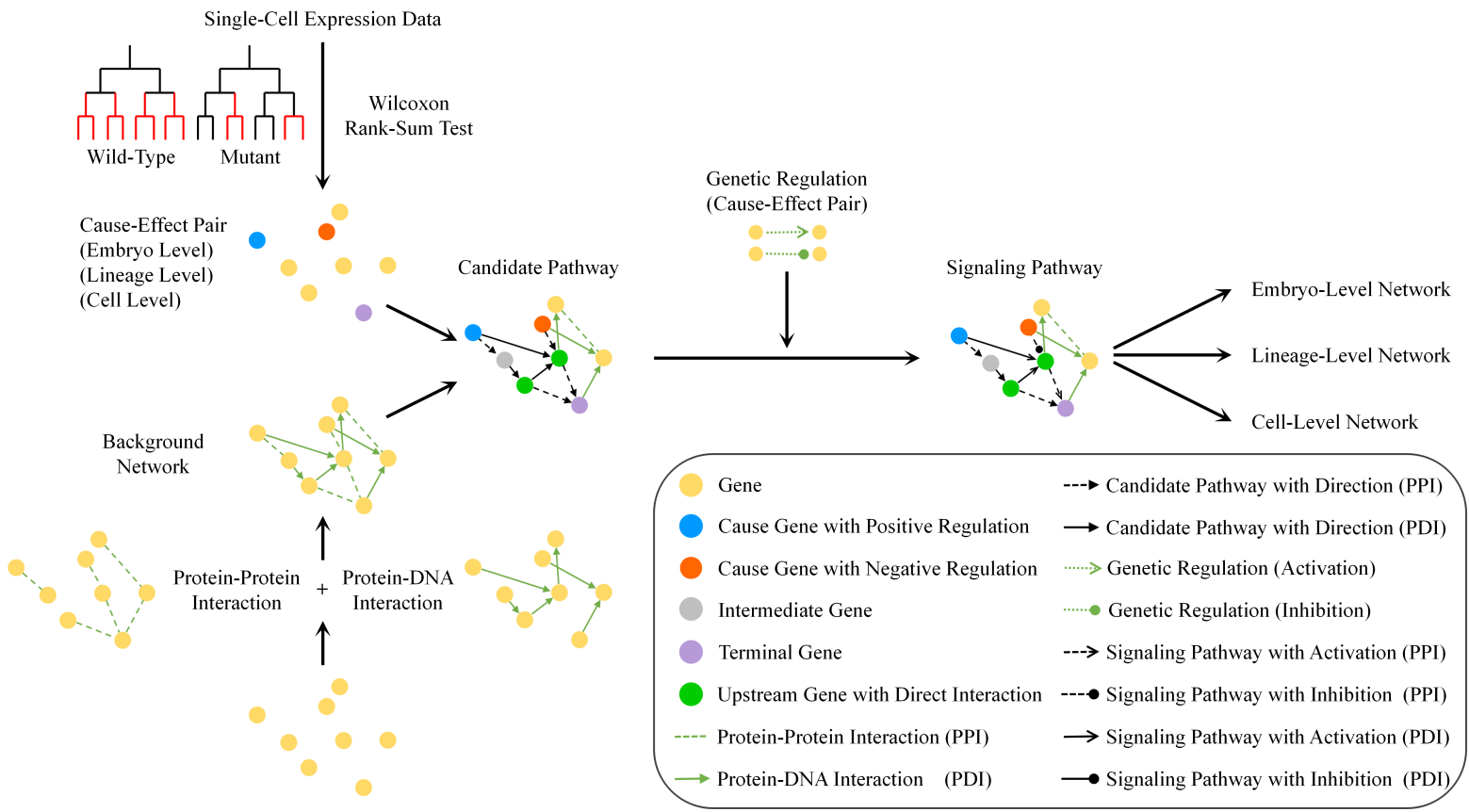


**Figure S3**


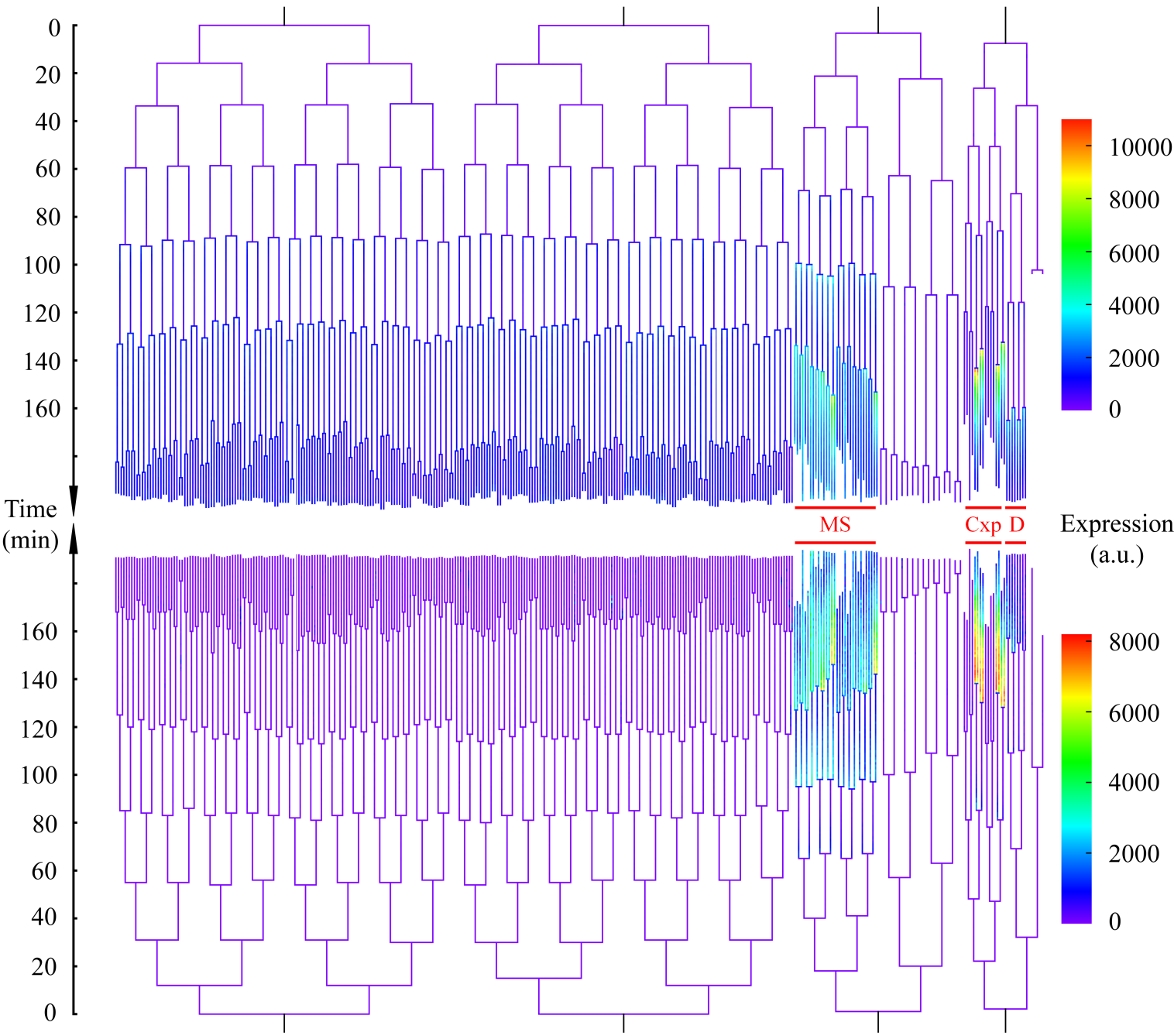


**Figure S4**


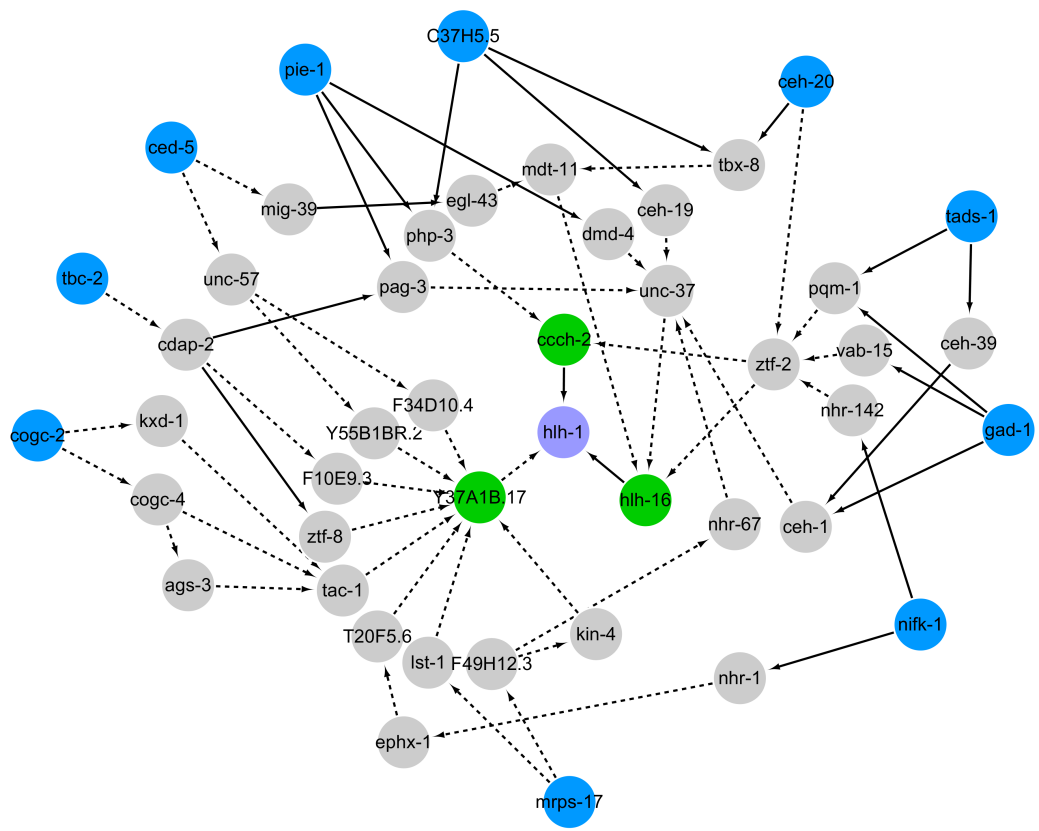


**Figure S5**


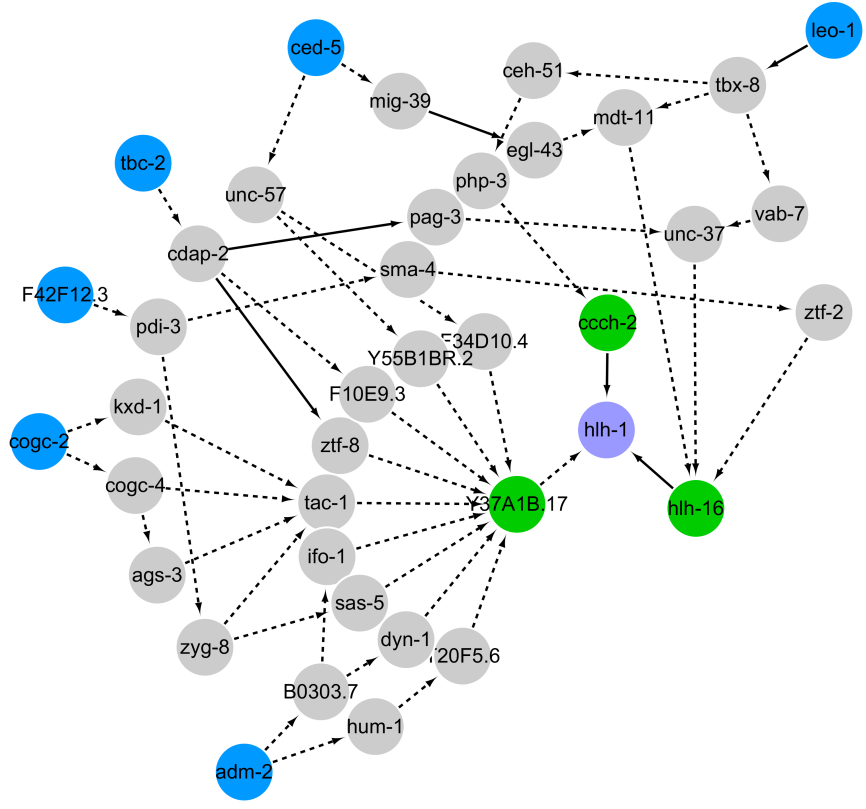


**Figure S6**


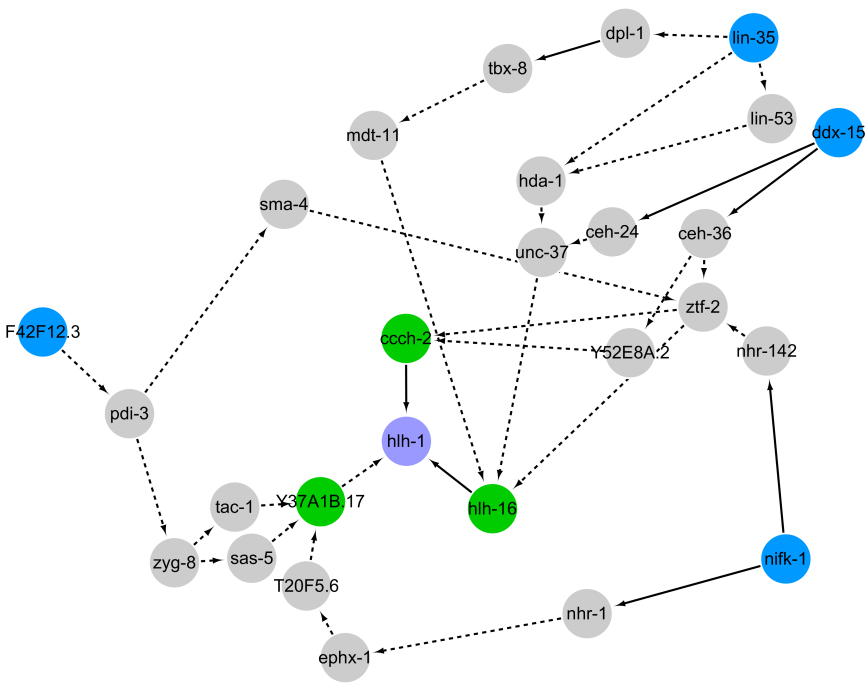


**Figure S7**


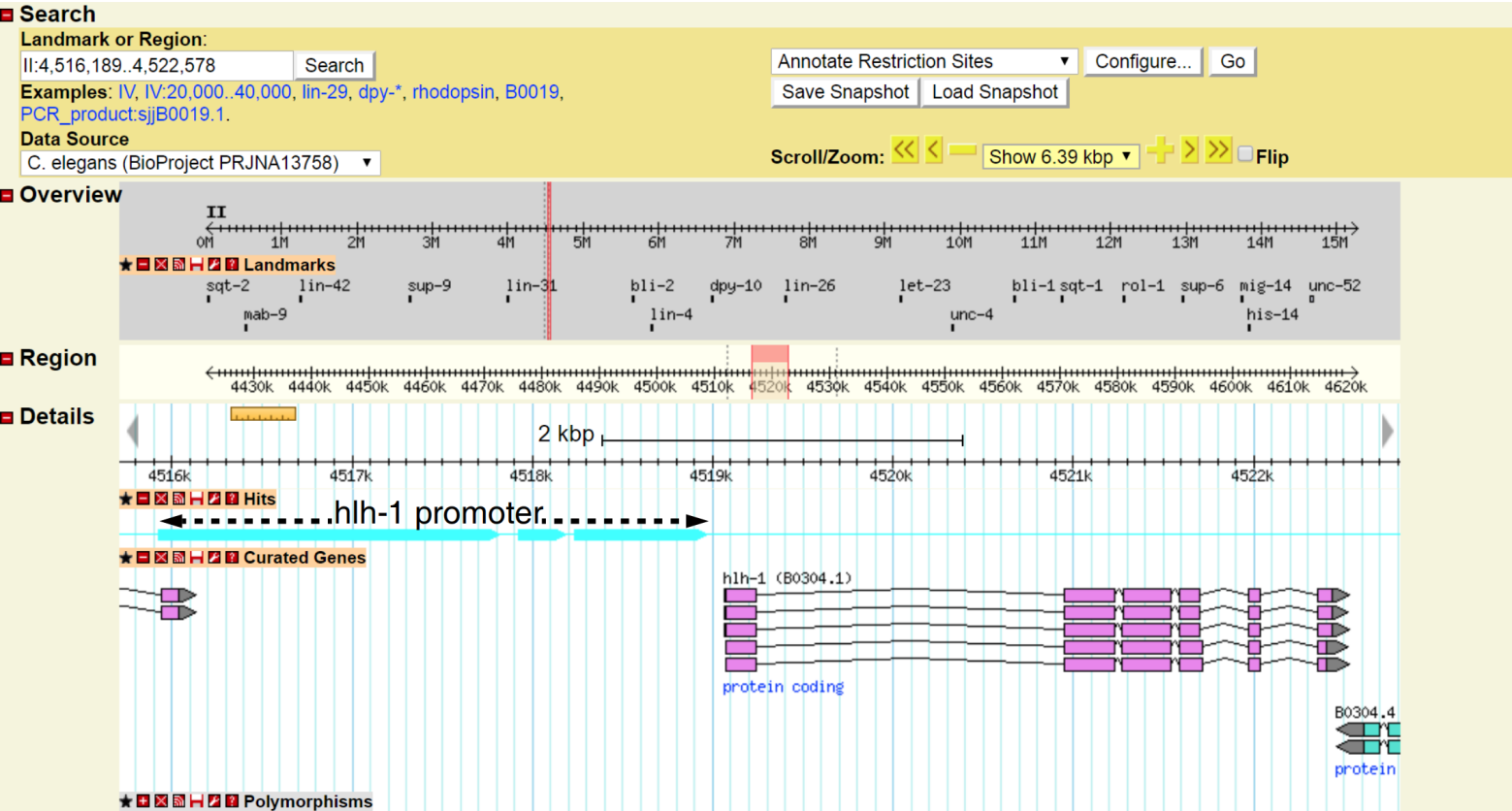


**Table S1**. Comparison of *hlh-1* expression intensity between cell groups (lineages/sublineages) coexisting at the same time.

| Cell Group | **MS16 (MSaxxx)** | AB64 | E8 | C8 | D4 |
| --- | --- | --- | --- | --- | --- |
| Expression (a.u.) | **1748 ± 158** | 832 ± 123 | 325 ± 7 | 419 ± 123 | 363 ± 42 |
| *p*-value | **/** | 4.6922×10^-6^ | 1.5540×10^-4^ | 1.5540×10^-4^ | 4.0000×10^-3^ |
| Cell Group | **MS16 (MSpxxx)** | AB64 | E8 | C8 | D4 |
| Expression (a.u.) | **1181 ± 104** | 832 ± 123 | 325 ± 7 | 419 ± 123 | 363 ± 42 |
| *p*-value | / | 9.218×10^-6^ | 1.5540×10^-4^ | 1.5540×10^-4^ | 4.0000×10^-3^ |
| Cell Group | **MS32 (MSaxxxx)** | AB128 | E8 | C16 (Caaxx) | C16 (Cpaxx) |
| Expression (a.u.) | **2977 ± 623** | 1091 ± 178 | 325 ± 7 | 496 ± 105 | 637 ± 164 |
| *p*-value | / | 7.7060×10^-11^ | 1.0084×10^-4^ | 2.9000×10^-3^ | 2.9000×10^-3^ |
| Cell Group | **MS32 (MSpxxxx)** | AB128 | E8 | C16 (Caaxx) | C16 (Cpaxx) |
| Expression (a.u.) | **1969 ± 521** | 1091 ± 178 | 325 ± 7 | 496 ± 105 | 637 ± 164 |
| *p*-value | / | 8.7469×10^-11^ | 1.0084×10^-4^ | 2.9000×10^-3^ | 2.9000×10^-3^ |
| Cell Group | **C16 (Capxx)** | AB128 | E8 | C16 (Caaxx) | C16 (Cpaxx) |
| Expression (a.u.) | **3800 ± 435** | 1091 ± 178 | 325 ± 7 | 496 ± 105 | 637 ± 164 |
| *p*-value | / | 6.9454×10^-11^ | 4.0000×10^-3^ | 2.8600×10^-2^ | 2.8600×10^-2^ |
| Cell Group | **C16 (Cppxx)** | AB128 | E8 | C16 (Caaxx) | C16 (Cpaxx) |
| Expression (a.u.) | **3246 ± 436** | 1091 ± 178 | 325 ± 7 | 496 ± 105 | 637 ± 164 |
| *p*-value | / | 6.9454×10^-11^ | 4.0000×10^-3^ | 2.8600×10^-2^ | 2.8600×10^-2^ |
| Cell Group | **D8 (Dxxx)** | AB256 | E8 | E16 | / |
| Expression (a.u.) | **1599 ± 194** | 1012 ± 208 | 325 ± 7 | 290 ± 35 | / |
| *p*-value | / | 3.5861×10^-6^ | 1.5540×10^-4^ | 1.0084×10^-4^ | / |

**Table S2**. Comparison of *hlh-1* expression intensity between high-expressing cell groups (lineages/sublineages).

| *p*-value | **MS16**  **(MSaxxx)** | *p*-value | **MS32**  **(MSaxxxx)** | *p*-value | **C16**  **(Cppxx)** | *p*-value | **D8**  **(Dxxx)** |
| --- | --- | --- | --- | --- | --- | --- | --- |
| **MS16**  **(MSpxxx)** | 1.5540×10^-4^ | **MS32**  **(MSpxxxx)** | 1.5203×10^-4^ | **C16**  **(Capxx)** | 0.1143 | **C16**  **(Cxpxx)** | 1.5540×10^-4^ |
| *p*-value | **MS32**  **(MSaxxxx)** | *p*-value | **MS32**  **(MSpxxxx)** | *p*-value | **D8**  **(Dxxx)** | *p*-value | **D8**  **(Dxxx)** |
| **C16**  **(Cxpxx)** | 0.0346 | **C16**  **(Cxpxx)** | 2.1151×10^-4^ | **MS32**  **(MSaxxxx)** | 1.0084×10^-4^ | **MS32**  **(MSpxxxx)** | 0.0537 |

Note: the *p*-values larger than 0.05 were painted blue, indicating no/little significant difference between the two groups.

**Table S3**. Experimental information about the 13 wild-type embryos.

| **Serial Number** | **File Name** | **Time Resolution**  **(min)** | **Last Time of**  **4-Cell Stage (min)** | **Ending Time of**  **~ 350-Cell Stage (min)** | **Cell Number at the Ending Time Point** | **Relative Global**  **Expression Intensity** |
| --- | --- | --- | --- | --- | --- | --- |
| 1 | 'CD120711hlh1p2.csv' | 1.54 | 12.33 | 223.54 | 359 | 1.0105 |
| 2 | 'CD120711hlh1p3.csv' | 1.54 | 15.42 | 223.54 | 359 | 0.9657 |
| 3 | 'CD120726hlh1p1.csv' | 1.54 | 9.25 | 238.96 | 361 | 1.2784 |
| 4 | 'CD120726hlh1p2.csv' | 1.54 | 6.17 | 231.25 | 357 | 1.0958 |
| 5 | 'CD120726hlh1p4.csv' | 1.54 | 9.25 | 231.25 | 354 | 1.3088 |
| 6 | 'CD140221hlh1p1.csv' | 1.41 | 15.49 | 211.25 | 359 | 0.9038 |
| 7 | 'CD140221hlh1p2.csv' | 1.41 | 11.27 | 218.29 | 357 | 0.7197 |
| 8 | 'CD140221hlh1p3.csv' | 1.41 | 25.35 | 225.33 | 359 | 0.8282 |
| 9 | 'CD140222hlh1p1.csv' | 1.41 | 18.31 | 232.38 | 357 | 1.2649 |
| 10 | 'CD140222hlh1p2.csv' | 1.41 | 14.08 | 204.21 | 359 | 0.8027 |
| 11 | 'CD140224hlh1p1.csv' | 1.41 | 14.08 | 204.21 | 359 | 0.8757 |
| 12 | 'CD140224hlh1p2.csv' | 1.41 | 12.68 | 197.17 | 359 | 1.1019 |
| 13 | 'CD140224hlh1p3.csv' | 1.41 | 8.45 | 197.17 | 361 | 0.8439 |

**Table S4**. Experimental information about the 133 RNAi-treated embryos.

| **Gene**  **Name** | **File Name** | **Time Resolution**  **(min)** | **Last Time of**  **4-Cell Stage (min)** | **Ending Time of**  **~ 350-Cell Stage (min)** | **Cell Number at the Ending Time Point** | **Relative Global**  **Expression Intensity** |
| --- | --- | --- | --- | --- | --- | --- |
| *'pie-1'* | 'CD120717hlh1pie1ip1.csv' | 1.54 | 1.54 | 185.00 | 355 | 0.2850 |
| *'pie-1'* | 'CD120717hlh1pie1ip2.csv' | 1.54 | 10.79 | 200.42 | 357 | 0.2599 |
| *'pie-1'* | 'CD120717hlh1pie1ip3.csv' | 1.54 | 1.54 | 185.00 | 368 | 0.2916 |
| *'C46G7.1'* | 'CD120727hlh1C46G7-1ip2.csv' | 1.54 | 7.71 | 223.54 | 353 | 1.1251 |
| *'C46G7.1'* | 'CD120727hlh1C46G7-1ip3.csv' | 1.54 | 10.79 | 215.83 | 350 | 1.3743 |
| *'C37H5.5'* | 'CD120731hlh1C37H5-5ip1.csv' | 1.54 | 6.17 | 200.42 | 357 | 0.5036 |
| *'C37H5.5'* | 'CD120731hlh1C37H5-5ip4.csv' | 1.54 | 18.50 | 231.25 | 345 | 0.7017 |
| *'par-2'* | 'CD120731hlh1par2ip2.csv' | 1.54 | 18.50 | 231.25 | 350 | 0.6019 |
| *'par-2'* | 'CD120731hlh1par2ip3.csv' | 1.54 | 18.50 | 215.83 | 394 | 0.6467 |
| *'hrdl-1'* | 'CD120802hlh1hrdl1ip3.csv' | 1.54 | 9.25 | 200.42 | 359 | 0.9220 |
| *'hrdl-1'* | 'CD120802hlh1hrdl1ip4.csv' | 1.54 | 16.96 | 215.83 | 358 | 0.8288 |
| *'lin-35'* | 'CD120802hlh1lin35ip3.csv' | 1.54 | 21.58 | 238.96 | 359 | 1.2311 |
| *'lin-35'* | 'CD120802hlh1lin35ip4.csv' | 1.54 | 23.13 | 231.25 | 349 | 0.8128 |
| *'C45G9.5'* | 'CD120804hlh1C45G9-5ip2.csv' | 1.54 | 4.63 | 238.96 | 356 | 1.9908 |
| *'C45G9.5'* | 'CD120804hlh1C45G9-5ip4.csv' | 1.54 | 20.04 | 238.96 | 368 | 1.0403 |
| *'vab-10'* | 'CD120804hlh1vab10ip1.csv' | 1.54 | 12.33 | 215.83 | 362 | 2.1505 |
| *'vab-10'* | 'CD120804hlh1vab10ip3.csv' | 1.54 | 10.79 | 231.25 | 321 | 1.5681 |
| *'wwp-1'* | 'CD120806hlh1wwp1ip1.csv' | 1.54 | 13.88 | 208.13 | 361 | 0.8898 |
| *'wwp-1'* | 'CD120806hlh1wwp1ip3.csv' | 1.54 | 10.79 | 231.25 | 412 | 1.1016 |
| *'ceh-27'* | 'CD120807hlh1ceh27ip2.csv' | 1.54 | 15.42 | 192.71 | 360 | 0.8097 |
| *'ceh-27'* | 'CD120807hlh1ceh27ip3.csv' | 1.54 | 3.08 | 231.25 | 393 | 0.7598 |
| *'nhr-25'* | 'CD120807hlh1nhr25ip1.csv' | 1.54 | 29.29 | 231.25 | 341 | 0.2273 |
| *'nhr-25'* | 'CD120807hlh1nhr25ip2.csv' | 1.54 | 3.08 | 208.13 | 359 | 0.9219 |
| *'ubc-12'* | 'CD120807hlh1ubc12ip2.csv' | 1.54 | 1.54 | 231.25 | 376 | 1.3597 |
| *'ubc-12'* | 'CD120807hlh1ubc12ip3.csv' | 1.54 | 12.33 | 231.25 | 349 | 2.2523 |
| *'lin-39'* | 'CD120811hlh1lin39ip1.csv' | 1.54 | 13.88 | 200.42 | 355 | 0.7096 |
| *'lin-39'* | 'CD120811hlh1lin39ip2.csv' | 1.54 | 20.04 | 223.54 | 365 | 0.8542 |
| *'C01F1.3'* | 'CD120814hlh1C01F1-3ip1.csv' | 1.54 | 24.67 | 231.25 | 363 | 1.2255 |
| *'C01F1.3'* | 'CD120814hlh1C01F1-3ip2.csv' | 1.54 | 16.96 | 208.13 | 355 | 0.9932 |
| *'C18A3.3'* | 'CD120814hlh1C18A3-3ip2.csv' | 1.54 | 3.08 | 208.13 | 372 | 1.0865 |
| *'C18A3.3'* | 'CD120814hlh1C18A3-3ip3.csv' | 1.54 | 7.71 | 200.42 | 359 | 1.1430 |
| *'nifk-1'* | 'CD120815hlh1T04B8-6ip1.csv' | 1.54 | 4.63 | 231.25 | 425 | 0.7846 |
| *'nifk-1'* | 'CD120815hlh1T04B8-6ip2.csv' | 1.54 | 10.79 | 208.13 | 359 | 0.5499 |
| *'Y45F10D.7'* | 'CD120815hlh1Y45F10D-ip2.csv' | 1.54 | 10.79 | 208.13 | 359 | 0.6515 |
| *'Y45F10D.7'* | 'CD120815hlh1Y45F10D-ip4.csv' | 1.54 | 38.54 | 308.33 | 358 | 1.5445 |
| *'ZK1098.1'* | 'CD120815hlh1ZK1098-1ip1.csv' | 1.54 | 15.42 | 215.83 | 366 | 1.4534 |
| *'ZK1098.1'* | 'CD120815hlh1ZK1098-1ip2.csv' | 1.54 | 13.88 | 215.83 | 358 | 0.8900 |
| *'gad-1'* | 'CD120816hlh1gad1ip1.csv' | 1.54 | 4.63 | 246.67 | 298 | 0.3561 |
| *'gad-1'* | 'CD120816hlh1gad1ip2.csv' | 1.54 | 3.08 | 246.67 | 271 | 0.4070 |
| *'vps-11'* | 'CD120816hlh1vps11ip1.csv' | 1.54 | 3.08 | 246.67 | 323 | 1.0323 |
| *'vps-11'* | 'CD120816hlh1vps11ip3.csv' | 1.54 | 30.83 | 254.38 | 360 | 0.8497 |
| *'ceh-20'* | 'CD120817hlh1ceh20ip1.csv' | 1.54 | 7.71 | 215.83 | 358 | 0.4363 |
| *'ceh-20'* | 'CD120817hlh1ceh20ip2.csv' | 1.54 | 13.88 | 208.13 | 357 | 0.7051 |
| *'ceh-6'* | 'CD120817hlh1ceh6ip1.csv' | 1.54 | 12.33 | 238.96 | 351 | 0.8808 |
| *'ceh-6'* | 'CD120817hlh1ceh6ip4.csv' | 1.54 | 13.88 | 231.25 | 372 | 0.6501 |
| *'enpl-1'* | 'CD120821hlh1T05E11-3ip3.csv' | 1.54 | 4.63 | 200.42 | 353 | 1.1683 |
| *'enpl-1'* | 'CD120821hlh1T05E11-3ip4.csv' | 1.54 | 3.08 | 200.42 | 357 | 1.1496 |
| *'glp-1'* | 'CD120822hlh1glp1ip1.csv' | 1.54 | 9.25 | 231.25 | 379 | 1.0017 |
| *'glp-1'* | 'CD120822hlh1glp1ip2.csv' | 1.54 | 23.13 | 231.25 | 356 | 1.3802 |
| *'mnat-1'* | 'CD120822hlh1mnat1ip2.csv' | 1.54 | 1.54 | 192.71 | 359 | 1.0489 |
| *'mnat-1'* | 'CD120822hlh1mnat1ip4.csv' | 1.54 | 4.63 | 231.25 | 413 | 0.9186 |
| *'C48E7.2'* | 'CD120823hlh1C48E7-2ip1.csv' | 1.54 | 13.88 | 215.83 | 361 | 1.2028 |
| *'C48E7.2'* | 'CD120823hlh1C48E7-2ip2.csv' | 1.54 | 20.04 | 215.83 | 364 | 0.6001 |
| *'B0238.11'* | 'CD120824hlh1B0238-11ip2.csv' | 1.54 | 13.88 | 208.13 | 358 | 1.0358 |
| *'B0238.11'* | 'CD120824hlh1B0238-11ip4.csv' | 1.54 | 7.71 | 215.83 | 364 | 1.1163 |
| *'par-1'* | 'CD120828hlh1par1ip2.csv' | 1.54 | 9.25 | 215.83 | 359 | 1.6821 |
| *'par-1'* | 'CD120828hlh1par1ip4.csv' | 1.54 | 1.54 | 200.42 | 361 | 1.3235 |
| *'rrp-1* | 'CD120830hlh1C47E12-7ip1.csv' | 1.54 | 13.88 | 215.83 | 352 | 0.3358 |
| *'rrp-1'* | 'CD120830hlh1C47E12-7ip2.csv' | 1.54 | 6.17 | 192.71 | 358 | 0.5494 |
| *'Y94H6A.5'* | 'CD120830hlh1Y94H6A-5ip1.csv' | 1.54 | 6.17 | 200.42 | 359 | 0.4791 |
| *'Y94H6A.5'* | 'CD120830hlh1Y94H6A-5ip3.csv' | 1.54 | 6.17 | 215.83 | 373 | 0.5142 |
| *'algn-11'* | 'CD120831hlh1B0361-8ip2.csv' | 1.54 | 1.54 | 215.83 | 383 | 0.6310 |
| *'algn-11'* | 'CD120831hlh1B0361-8ip3.csv' | 1.54 | 10.79 | 200.42 | 358 | 0.4908 |
| *'mrps-31'* | 'CD120831hlh1C32A3-2ip1.csv' | 1.54 | 7.71 | 231.25 | 373 | 1.4085 |
| *'mrps-31'* | 'CD120831hlh1C32A3-2ip2.csv' | 1.54 | 4.63 | 208.13 | 358 | 1.1590 |
| *'C32E12.4'* | 'CD120901hlh1C32E12-4ip1.csv' | 1.54 | 13.88 | 223.54 | 371 | 0.5832 |
| *'C32E12.4'* | 'CD120901hlh1C32E12-4ip2.csv' | 1.54 | 6.17 | 200.42 | 360 | 0.7811 |
| *'ddx-15'* | 'CD120901hlh1F56D2-6ip2.csv' | 1.54 | 13.88 | 223.54 | 357 | 0.7454 |
| *'ddx-15'* | 'CD120901hlh1F56D2-6ip3.csv' | 1.54 | 9.25 | 215.83 | 359 | 0.7516 |
| *'cdc-25.2'* | 'CD120903hlh1cdc-25-2ip3.csv' | 1.54 | 9.25 | 231.25 | 199 | 1.1102 |
| *'cdc-25.2'* | 'CD120903hlh1cdc-25-2ip4.csv' | 1.54 | 16.96 | 277.50 | 212 | 1.6545 |
| *'cdc-6'* | 'CD120904hlh1cdc6ip2.csv' | 1.54 | 12.33 | 231.25 | 329 | 0.7888 |
| *'cdc-6'* | 'CD120904hlh1cdc6ip3.csv' | 1.54 | 15.42 | 246.67 | 228 | 0.9040 |
| *'mrps-17'* | 'CD120905hlh1C05D11-1ip1.csv' | 1.54 | 7.71 | 200.42 | 359 | 0.4891 |
| *'mrps-17'* | 'CD120905hlh1C05D11-1ip3.csv' | 1.54 | 12.33 | 223.54 | 372 | 0.6807 |
| *'gly-17'* | 'CD120905hlh1gly17ip1.csv' | 1.54 | 6.17 | 208.13 | 376 | 0.7295 |
| *'gly-17'* | 'CD120905hlh1gly17ip2.csv' | 1.54 | 6.17 | 231.25 | 359 | 1.4186 |
| *'rap-2'* | 'CD120906hlh1rap2ip2.csv' | 1.54 | 10.79 | 200.42 | 360 | 0.7346 |
| *'rap-2'* | 'CD120906hlh1rap2ip3.csv' | 1.54 | 1.54 | 208.13 | 373 | 0.7332 |
| *'B0035.3'* | 'CD120907hlh1B0035-3ip3.csv' | 1.54 | 7.71 | 215.83 | 373 | 0.5425 |
| *'B0035.3'* | 'CD120907hlh1B0035-3ip4.csv' | 1.54 | 10.79 | 200.42 | 357 | 0.4515 |
| *'arx-1'* | 'CD120910hlh1arx1ip1.csv' | 1.54 | 20.04 | 215.83 | 358 | 1.4153 |
| *'arx-1'* | 'CD120910hlh1arx1ip4.csv' | 1.54 | 13.88 | 231.25 | 357 | 0.6754 |
| *'efl-1'* | 'CD120911hlh1efl1ip2.csv' | 1.54 | 7.71 | 200.42 | 348 | 1.0819 |
| *'efl-1'* | 'CD120911hlh1efl1ip3.csv' | 1.54 | 6.17 | 200.42 | 360 | 0.7373 |
| *'kpc-1'* | 'CD120911hlh1kpc1ip2.csv' | 1.54 | 3.08 | 208.13 | 376 | 0.6499 |
| *'kpc-1'* | 'CD120911hlh1kpc1ip3.csv' | 1.54 | 20.04 | 215.83 | 357 | 0.8942 |
| *'nra-4'* | 'CD120912hlh1nra4ip2.csv' | 1.54 | 21.58 | 231.25 | 355 | 0.7124 |
| *'nra-4'* | 'CD120912hlh1nra4ip4.csv' | 1.54 | 21.58 | 223.54 | 354 | 0.6636 |
| *'magu-2'* | 'CD120912hlh1tag117ip1.csv' | 1.54 | 3.08 | 215.83 | 366 | 1.1248 |
| *'magu-2'* | 'CD120912hlh1tag117ip2.csv' | 1.54 | 13.88 | 231.25 | 359 | 0.9197 |
| *'C50B6.7'* | 'CD120913hlh1C50B6-7ip1.csv' | 1.54 | 13.88 | 223.54 | 380 | 0.4431 |
| *'C50B6.7'* | 'CD120913hlh1C50B6-7ip3.csv' | 1.54 | 15.42 | 208.13 | 359 | 0.5093 |
| *'hars-1'* | 'CD120913hlh1hars1ip1.csv' | 1.54 | 1.54 | 262.08 | 158 | 2.5145 |
| *'hars-1'* | 'CD120913hlh1hars1ip2.csv' | 1.54 | 12.33 | 215.83 | 361 | 0.8886 |
| *'rod-1'* | 'CD120913hlh1rod1ip3.csv' | 1.54 | 4.63 | 215.83 | 351 | 1.0427 |
| *'rod-1'* | 'CD120913hlh1rod1ip4.csv' | 1.54 | 1.54 | 200.42 | 355 | 0.9140 |
| *'tads-1'* | 'CD120914hlh1C01A2-5ip1.csv' | 1.54 | 9.25 | 231.25 | 358 | 0.2923 |
| *'tads-1'* | 'CD120914hlh1C01A2-5ip3.csv' | 1.54 | 13.88 | 238.96 | 350 | 0.2963 |
| *'hst-1'* | 'CD120914hlh1hst1ip1.csv' | 1.54 | 3.08 | 208.13 | 360 | 0.6035 |
| *'hst-1'* | 'CD120914hlh1hst1ip4.csv' | 1.54 | 21.58 | 246.67 | 277 | 1.2305 |
| *'tbc-2'* | 'CD120914hlh1tbc2ip1.csv' | 1.54 | 7.71 | 215.83 | 352 | 0.5170 |
| *'tbc-2'* | 'CD120914hlh1tbc2ip3.csv' | 1.54 | 13.88 | 215.83 | 355 | 0.4941 |
| *'pitr-3'* | 'CD120915hlh1B0222-3ip1.csv' | 1.54 | 4.63 | 192.71 | 360 | 0.5356 |
| *'pitr-3'* | 'CD120915hlh1B0222-3ip4.csv' | 1.54 | 18.50 | 215.83 | 335 | 0.7011 |
| *'wsp-1'* | 'CD120917hlh1wsp1ip1.csv' | 1.54 | 1.54 | 200.42 | 335 | 0.8855 |
| *'wsp-1'* | 'CD120917hlh1wsp1ip2.csv' | 1.54 | 12.33 | 215.83 | 358 | 0.8131 |
| *'leo-1'* | 'CD120919hlh1B0035-11ip2.csv' | 1.54 | 24.67 | 246.67 | 351 | 0.6414 |
| *'leo-1'* | 'CD120919hlh1B0035-11ip3.csv' | 1.54 | 3.08 | 238.96 | 356 | 0.5586 |
| *'cogc-2'* | 'CD120919hlh1C06G3-10ip3.csv' | 1.54 | 6.17 | 231.25 | 349 | 0.6604 |
| *'cogc-2'* | 'CD120919hlh1C06G3-10ip4.csv' | 1.54 | 1.54 | 200.42 | 355 | 0.4915 |
| *'cogc-2'* | 'CD120920hlh1cogc-2ip2.csv' | 1.54 | 1.54 | 223.54 | 359 | 0.4589 |
| *'cogc-2'* | 'CD120920hlh1cogc-2ip3.csv' | 1.54 | 18.50 | 246.67 | 348 | 0.5727 |
| *'abt-2'* | 'CD120920hlh1abt-2ip1.csv' | 1.54 | 9.25 | 231.25 | 357 | 1.0025 |
| *'abt-2'* | 'CD120920hlh1abt-2ip3.csv' | 1.54 | 15.42 | 215.83 | 337 | 0.8230 |
| *'gly-16'* | 'CD120920hlh1gly16ip1.csv' | 1.54 | 21.58 | 238.96 | 351 | 0.8697 |
| *'gly-16'* | 'CD120920hlh1gly16ip2.csv' | 1.54 | 10.79 | 231.25 | 342 | 0.8363 |
| *'elp-1'* | 'CD120921hlh1elp1ip1.csv' | 1.54 | 1.54 | 215.83 | 342 | 0.9058 |
| *'elp-1'* | 'CD120921hlh1elp1ip2.csv' | 1.54 | 3.08 | 200.42 | 354 | 0.7624 |
| *'sur-6'* | 'CD120922hlh1sur6ip3.csv' | 1.54 | 3.08 | 215.83 | 364 | 1.0665 |
| *'sur-6'* | 'CD120922hlh1sur6ip4.csv' | 1.54 | 10.79 | 200.42 | 352 | 0.6499 |
| *'F42F12.3'* | 'CD120925hlh1F42F12-3ip1.csv' | 1.54 | 1.54 | 223.54 | 349 | 0.3297 |
| *'F42F12.3'* | 'CD120925hlh1F42F12-3ip2.csv' | 1.54 | 1.54 | 200.42 | 337 | 0.5484 |
| *'pept-2'* | 'CD120925hlh1pept2ip2.csv' | 1.54 | 4.63 | 208.13 | 350 | 0.6913 |
| *'pept-2'* | 'CD120925hlh1pept2ip3.csv' | 1.54 | 10.79 | 223.54 | 356 | 1.0695 |
| *'nap-1'* | 'CD120926hlh1D2096-8ip1.csv' | 1.54 | 27.75 | 238.96 | 357 | 0.5734 |
| *'nap-1'* | 'CD120926hlh1D2096-8ip2.csv' | 1.54 | 7.71 | 215.83 | 354 | 0.8385 |
| *'alg-1'* | 'CD120926hlh1alg1ip2.csv' | 1.54 | 12.33 | 223.54 | 361 | 1.0355 |
| *'alg-1'* | 'CD120926hlh1alg1ip3.csv' | 1.54 | 10.79 | 203.50 | 344 | 0.8649 |
| *'adm-2'* | 'CD120927hlh1adm2ip1.csv' | 1.54 | 6.17 | 215.83 | 356 | 0.6431 |
| *'adm-2'* | 'CD120927hlh1adm2ip2.csv' | 1.54 | 10.79 | 231.25 | 350 | 0.6837 |
| *'ced-5'* | 'CD120927hlh1ced5ip1.csv' | 1.54 | 24.67 | 231.25 | 347 | 0.4470 |
| *'ced-5'* | 'CD120927hlh1ced5ip3.csv' | 1.54 | 12.33 | 215.83 | 355 | 0.6160 |

**Table S5**. The three most probable pathways inferred with PPI, PDI, GI and RNAi data.

| **Selected Pathway** | **Interaction Type of Data** | **Regulatory Form**  **(0, negative; 1, positive)** |
| --- | --- | --- |
| *leo-1 tbx-8 mdt-11 hlh-16 hlh-1* | PDI PPI PPI PDI | 0\1,0\1,0\1,0\1 |
| *leo-1 tbx-8 ceh-51 php-3 ccch-2 hlh-1* | PDI PPI PPI PPI PDI | 0\1,0\1,0\1,0\1,0\1 |
| *leo-1 tbx-8 vab-7 unc-37 hlh-16 hlh-1* | PDI PPI PPI PPI PDI | 0\1,1,0\1,0\1,0\1 |
| *tads-1 pqm-1 ztf-2 hlh-16 hlh-1* | PDI PPI PPI PDI | 0\1,0\1,0\1,0\1 |
| *tads-1 pqm-1 ztf-2 ccch-2 hlh-1* | PDI PPI PPI PDI | 0\1,0\1,0\1,0\1 |
| *tads-1 ceh-39 ceh-1 unc-37 hlh-16 hlh-1* | PDI PDI PPI PPI PDI | 0\1,0\1,0\1,0\1,0\1 |
| *ced-5 unc-57 F34D10.4 Y37A1B.17 hlh-1* | PPI PPI PPI PPI | 0\1,0\1,0\1,0\1 |
| *ced-5 unc-57 Y55B1BR.2 Y37A1B.17 hlh-1* | PPI PPI PPI PPI | 0\1,0\1,0\1,0\1 |
| *ced-5 mig-39 egl-43 mdt-11 hlh-16 hlh-1* | PPI PDI PPI PPI PDI | 0\1,0\1,0\1,0\1,0\1 |
| *adm-2 B0303.7 ifo-1 Y37A1B.17 hlh-1* | PPI PPI PPI PPI | 0\1,0\1,0\1,0\1 |
| *adm-2 hum-1 T20F5.6 Y37A1B.17 hlh-1* | PPI PPI PPI PPI | 0\1,0\1,0\1,0\1 |
| *adm-2 B0303.7 dyn-1 Y37A1B.17 hlh-1* | PPI PPI PPI PPI | 0\1,0\1,0\1,0\1 |
| *mrps-17 lst-1 Y37A1B.17 hlh-1* | PPI PPI PPI | 0\1,0\1,0\1 |
| *mrps-17 F49H12.3 kin-4 Y37A1B.17 hlh-1* | PPI PPI PPI PPI | 0\1,0\1,0\1,0\1 |
| *mrps-17 F49H12.3 nhr-67 unc-37 hlh-16 hlh-1* | PPI PPI PPI PPI PDI | 0\1,0\1,0\1,0\1,0\1 |
| *cogc-2 cogc-4 tac-1 Y37A1B.17 hlh-1* | PPI PPI PPI PPI | 0\1,0\1,0\1,0\1 |
| *cogc-2 kxd-1 tac-1 Y37A1B.17 hlh-1* | PPI PPI PPI PPI | 0\1,0\1,0\1,0\1 |
| *cogc-2 cogc-4 ags-3 tac-1 Y37A1B.17 hlh-1* | PPI PPI PPI PPI PPI | 0\1,0\1,0\1,0\1,0\1 |
| *lin-39 ztf-2 ccch-2 hlh-1* | PPI PPI PDI | 0\1,0\1,0\1 |
| *lin-39 tbx-9 ztf-2 ccch-2 hlh-1* | PDI PPI PPI PDI | 0\1,0\1,0\1,0\1 |
| *lin-39 ceh-20 ztf-2 ccch-2 hlh-1* | PDI PPI PPI PDI | 1,0\1,0\1,0\1 |
| *mrps-31 dcr-1 hip-1 Y37A1B.17 hlh-1* | PPI PPI PPI PPI | 0\1,0\1,0\1,0\1 |
| *mrps-31 dcr-1 cyn-6 php-3 ccch-2 hlh-1* | PPI PPI PDI PPI PDI | 0\1,0\1,0\1,0\1,0\1 |
| *mrps-31 dcr-1 tiar-2 php-3 ccch-2 hlh-1* | PPI PPI PDI PPI PDI | 0\1,0\1,0\1,0\1,0\1 |
| *lin-35 hda-1 unc-37 hlh-16 hlh-1* | PPI PPI PPI PDI | 0\1,0\1,0\1,0\1 |
| *lin-35 dpl-1 tbx-8 mdt-11 hlh-16 hlh-1* | PPI PDI PPI PPI PDI | 0\1,0\1,0\1,0\1,0\1 |
| *lin-35 lin-53 hda-1 unc-37 hlh-16 hlh-1* | PPI PPI PPI PPI PDI | 0\1,0\1,0\1,0\1,0\1 |
| *C37H5.5 php-3 ccch-2 hlh-1* | PDI PPI PDI | 0\1,0\1,0\1 |
| *C37H5.5 ceh-19 unc-37 hlh-16 hlh-1* | PDI PPI PPI PDI | 0\1,0\1,0\1,0\1 |
| *C37H5.5 tbx-8 mdt-11 hlh-16 hlh-1* | PDI PPI PPI PDI | 0\1,0\1,0\1,0\1 |
| *cdc-6 ima-3 arrd-13 php-3 ccch-2 hlh-1* | PPI PPI PDI PPI PDI | 0\1,0\1,0\1,0\1,0\1 |
| *cdc-6 ima-3 lag-1 ztf-8 Y37A1B.17 hlh-1* | PPI PPI PDI PPI PPI | 0\1,0\1,0\1,0\1,0\1 |
| *cdc-6 ima-3 arrd-13 ztf-8 Y37A1B.17 hlh-1* | PPI PPI PDI PPI PPI | 0\1,0\1,0\1,0\1,0\1 |
| *C45G9.5 vha-12 mig-5 php-3 ccch-2 hlh-1* | PPI PDI PDI PPI PDI | 0\1,0\1,0\1,0\1,0\1 |
| *C45G9.5 vha-12 nhr-49 ztf-2 ccch-2 hlh-1* | PPI PDI PPI PPI PDI | 0\1,0\1,0\1,0\1,0\1 |
| *C45G9.5 vha-12 nhr-49 ztf-2 hlh-16 hlh-1* | PPI PDI PPI PPI PDI | 0\1,0\1,0\1,0\1,0\1 |
| *nap-1 nhr-72 mig-5 php-3 ccch-2 hlh-1* | PDI PDI PDI PPI PDI | 0\1,0\1,0\1,0\1,0\1 |
| *nap-1 sma-3 mig-5 php-3 ccch-2 hlh-1* | PPI PDI PDI PPI PDI | 0\1,0\1,0\1,0\1,0\1 |
| *nap-1 nhr-72 lin-39 ztf-2 ccch-2 hlh-1* | PDI PDI PPI PPI PDI | 0\1,0\1,0\1,0\1,0\1 |
| *nhr-25 peb-1 hlh-27 ztf-2 ccch-2 hlh-1* | PDI PDI PDI PPI PDI | 0\1,0\1,0\1,0\1,0\1 |
| *nhr-25 hlh-27 mig-5 php-3 ccch-2 hlh-1* | PDI PDI PDI PPI PDI | 0\1,0\1,0\1,0\1,0\1 |
| *nhr-25 hlh-27 ztf-2 ccch-2 hlh-1* | PDI PDI PPI PDI | 0\1,0\1,0\1,0\1 |
| *ceh-20 tbx-8 mdt-11 hlh-16 hlh-1* | PDI PPI PPI PDI | 0\1,0\1,0\1,0\1 |
| *ceh-20 ztf-2 hlh-16 hlh-1* | PPI PPI PDI | 0\1,0\1,0\1 |
| *ceh-20 ztf-2 ccch-2 hlh-1* | PPI PPI PDI | 0\1,0\1,0\1 |
| *F42F12.3 pdi-3 zyg-8 tac-1 Y37A1B.17 hlh-1* | PPI PPI PPI PPI PPI | 0\1,0\1,0\1,0\1,0\1 |
| *F42F12.3 pdi-3 zyg-8 sas-5 Y37A1B.17 hlh-1* | PPI PPI PPI PPI PPI | 0\1,0\1,0\1,0\1,0\1 |
| *F42F12.3 pdi-3 sma-4 ztf-2 hlh-16 hlh-1* | PPI PPI PPI PPI PDI | 0\1,0\1,0\1,0\1,0\1 |
| *ddx-15 ceh-36 Y52E8A.2 ccch-2 hlh-1* | PDI PPI PPI PDI | 0\1,0\1,0\1,0\1 |
| *ddx-15 ceh-36 ztf-2 hlh-16 hlh-1* | PDI PPI PPI PDI | 0\1,0\1,0\1,0\1 |
| *ddx-15 ceh-24 unc-37 hlh-16 hlh-1* | PDI PPI PPI PDI | 0\1,0\1,0\1,0\1 |
| *par-2 lmn-1 klp-11 ifb-1 Y37A1B.17 hlh-1* | PPI PPI PPI PPI PPI | 0\1,0\1,0\1,0\1,0\1 |
| *par-2 lmn-1 tac-1 Y37A1B.17 hlh-1* | PPI PPI PPI PPI | 0\1,0\1,0\1,0\1 |
| *par-2 lmn-1 ztf-8 Y37A1B.17 hlh-1* | PPI PPI PPI PPI | 0\1,0\1,0\1,0\1 |
| *par-1 lev-11 unc-37 hlh-16 hlh-1* | PPI PPI PPI PDI | 0\1,0\1,0\1,0\1 |
| *par-1 let-99 tbx-8 mdt-11 hlh-16 hlh-1* | PPI PDI PPI PPI PDI | 0,0\1,0\1,0\1,0\1 |
| *par-1 let-99 pqm-1 ztf-2 hlh-16 hlh-1* | PPI PDI PPI PPI PDI | 0,0\1,0\1,0\1,0\1 |
| *ceh-6 klf-2 mig-5 php-3 ccch-2 hlh-1* | PDI PDI PDI PPI PDI | 0\1,0\1,0\1,0\1,0\1 |
| *ceh-6 wdr-5.1 mig-5 php-3 ccch-2 hlh-1* | PPI PDI PDI PPI PDI | 0\1,0\1,0\1,0\1,0\1 |
| *ceh-6 zip-7 mig-5 php-3 ccch-2 hlh-1* | PPI PDI PDI PPI PDI | 0\1,0\1,0\1,0\1,0\1 |
| *vps-11 vab-15 ztf-2 ccch-2 hlh-1* | PDI PPI PPI PDI | 0\1,0\1,0\1,0\1 |
| *vps-11 egl-5 lsy-2 hif-1 ccch-2 hlh-1* | PDI PDI PPI PPI PDI | 0\1,0\1,0\1,0\1,0\1 |
| *vps-11 egl-5 lsy-2 ztf-2 ccch-2 hlh-1* | PDI PDI PPI PPI PDI | 0\1,0\1,0\1,0\1,0\1 |
| *ubc-12 tbx-8 mdt-11 hlh-16 hlh-1* | PDI PPI PPI PDI | 0\1,0\1,0\1,0\1 |
| *ubc-12 ceh-9 unc-37 hlh-16 hlh-1* | PDI PPI PPI PDI | 0\1,0\1,0\1,0\1 |
| *ubc-12 dmd-3 ztf-2 hlh-16 hlh-1* | PDI PPI PPI PDI | 0\1,0\1,0\1,0\1 |
| *nifk-1 nhr-142 ztf-2 hlh-16 hlh-1* | PDI PPI PPI PDI | 0\1,0\1,0\1,0\1 |
| *nifk-1 nhr-1 ephx-1 T20F5.6 Y37A1B.17 hlh-1* | PDI PPI PPI PPI PPI | 0\1,0\1,0\1,0\1,0\1 |
| *nifk-1 nhr-142 ztf-2 ccch-2 hlh-1* | PDI PPI PPI PDI | 0\1,0\1,0\1,0\1 |
| *enpl-1 daf-19 dmd-3 ztf-2 ccch-2 hlh-1* | PDI PDI PPI PPI PDI | 0\1,0\1,0\1,0\1,0\1 |
| *enpl-1 daf-19 dmd-3 ztf-2 hlh-16 hlh-1* | PDI PDI PPI PPI PDI | 0\1,0\1,0\1,0\1,0\1 |
| *enpl-1 sgt-1 tomm-20 php-3 ccch-2 hlh-1* | PPI PPI PDI PPI PDI | 0\1,0\1,0\1,0\1,0\1 |
| *gad-1 ceh-1 unc-37 hlh-16 hlh-1* | PDI PPI PPI PDI | 0\1,0\1,0\1,0\1 |
| *gad-1 pqm-1 ztf-2 hlh-16 hlh-1* | PDI PPI PPI PDI | 0\1,0\1,0\1,0\1 |
| *gad-1 vab-15 ztf-2 hlh-16 hlh-1* | PDI PPI PPI PDI | 0\1,0\1,0\1,0\1 |
| *pie-1 php-3 ccch-2 hlh-1* | PDI PPI PDI | 0\1,0\1,0\1 |
| *pie-1 dmd-4 unc-37 hlh-16 hlh-1* | PDI PPI PPI PDI | 0\1,0\1,0\1,0\1 |
| *pie-1 pag-3 unc-37 hlh-16 hlh-1* | PDI PPI PPI PDI | 0\1,0\1,0\1,0\1 |
| *wwp-1 vab-3 mig-5 php-3 ccch-2 hlh-1* | PPI PDI PDI PPI PDI | 0\1,0\1,0\1,0\1,0\1 |
| *wwp-1 nhr-111 mig-5 php-3 ccch-2 hlh-1* | PPI PDI PDI PPI PDI | 0\1,0\1,0\1,0\1,0\1 |
| *wwp-1 ubc-18 tbx-9 ztf-2 ccch-2 hlh-1* | PPI PDI PPI PPI PDI | 0\1,0\1,0\1,0\1,0\1 |
| *vab-10 mua-6 dsh-2 T20F5.6 Y37A1B.17 hlh-1* | PPI PPI PPI PPI PPI | 0\1,0\1,0\1,0\1,0\1 |
| *vab-10 mua-6 mig-5 php-3 ccch-2 hlh-1* | PPI PPI PDI PPI PDI | 0\1,0\1,0\1,0\1,0\1 |
| *vab-10 mua-6 dsh-2 sas-5 Y37A1B.17 hlh-1* | PPI PPI PPI PPI PPI | 0\1,0\1,0\1,0\1,0\1 |
| *tbc-2 cdap-2 F10E9.3 Y37A1B.17 hlh-1* | PPI PPI PPI PPI | 0\1,0\1,0\1,0\1 |
| *tbc-2 cdap-2 ztf-8 Y37A1B.17 hlh-1* | PPI PDI PPI PPI | 0\1,0\1,0\1,0\1 |
| *tbc-2 cdap-2 pag-3 unc-37 hlh-16 hlh-1* | PPI PDI PPI PPI PDI | 0\1,0\1,0\1,0\1,0\1 |
